# Targeted Cancer Therapy Using Compounds Activated by Light

**DOI:** 10.3390/cancers13133237

**Published:** 2021-06-29

**Authors:** Petra Dunkel, Janez Ilaš

**Affiliations:** 1 Department of Organic Chemistry, Semmelweis University, 1092 Budapest, Hungary; dunkel.petra@pharma.semmelweis-univ.hu; 2 Faculty of Pharmacy, University of Ljubljana, 1000 Ljubljana, Slovenia

**Keywords:** photocages, photoswitches, photopharmacology, light-activatable materials, photoresponsive drug delivery systems, azobenzenes

## Abstract

**Simple Summary:**

Cancer is among the leading causes of death, and cancer therapy suffers from many drawbacks, the lack of selectivity being most noteworthy. In this review, we present innovative approaches in the discovery of novel anticancer compounds, which can use light activation to achieve more potent cancer therapy with fewer side effects. We describe recent approaches to prepare photocages and photoswitches and obstacles that photopharmacology must overcome to achieve clinical use.

**Abstract:**

Cancer chemotherapy is affected by a modest selectivity and toxic side effects of pharmacological interventions. Among novel approaches to overcome this limitation and to bring to therapy more potent and selective agents is the use of light for selective activation of anticancer compounds. In this review, we focus on the anticancer applications of two light-activated approaches still in the experimental phase: photoremovable protecting groups (“photocages”) and photoswitches. We describe the structural considerations behind the development of novel compounds and the plethora of assays used to confirm whether the photochemical and pharmacological properties are meeting the stringent criteria for an efficient in vivo light-dependent activation. Despite its immense potential, light activation brings many challenges, and the complexity of the task is very demanding. Currently, we are still deeply in the phase of pharmacological tools, but the vivid research and rapid development bring the light of hope for potential clinical use.

## 1. Introduction

**Figure 1:**
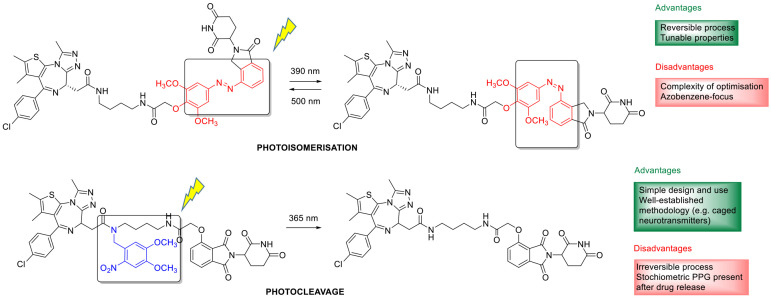
Photocaged vs. photoswitchable pharmacological agents, with the respective photoactivated transformations.

Cancer chemotherapy is affected by a modest selectivity and toxic side effects of pharmacological interventions [1]. Therefore, besides finding novel, efficient, targeted antitumor agents, developing innovative solutions for endowing cancer chemotherapeutics with a more selective, localized effect is of paramount importance. Applying various internal (e.g., pH, redox environment, enzymatic processes) or external triggers (e.g., ultrasound, magnetic field, light irradiation) for on-site activation of stimuli-sensitive drug molecules or drug delivery systems (as, e.g., liposomes, micelles, polymeric or metal nanoparticles, dendrimers) [2,3,4] has long been in the forefront of research interest. Of the potential physical stimuli, light is particular from several aspects, from its orthogonality with biological systems to the precise control over the wavelength and the irradiation dose. Light activation is independent of the properties of the tumor environment as well, allowing to target a variety of tumors. Light-activated approaches made their way into clinical use already in the 1990s, with the approval of the first photodynamic therapy regimes [5]. Photodynamic therapy (PDT) is based on the interaction of photosensitizer agents with light to produce singlet oxygen and superoxide in the presence of molecular oxygen and the local cytotoxic effect of these species. The discussion of PDT is beyond the scope of the present review, that will focus on the anticancer applications of two further light-activated approaches still in the experimental phase: photoremovable protecting groups (“photocages”) and photoswitches (illustrated in Figure 1 by the examples of a photocaged vs. a photoswitchable agent). The concept of photopharmacology was recently introduced to describe the area dealing with photocontrollable molecules for therapy. In this review, we focus on small-molecule approaches, where a parent drug itself is rendered photoactivatable via structural modifications, and the approach is backed up by in cellulo assays. However, more complex drug delivery systems could also be rendered light-responsive by judiciously designing photoactivatable units into their structure [6,7]. For such approaches and designs where photoactivation is used for controlling targeting or cell penetration [8,9] (i.e., not directly the drug activity), we refer to the respective reports and reviews (e.g., (upconversion) nanoparticles [10,11,12,13,14,15], organic nanoparticles [16], chitosan nanoparticles [17], gold nanoparticles [18], photocaged folate nanoconjugates [19], two-photon nanoimpellers [20], photoresponsive antibody-drug conjugates [21,22]).

## 2. Irreversible Activation with Light: Photoremovable Protecting Groups (“Photocages”) for Antitumor Applications

**Figure 2:**
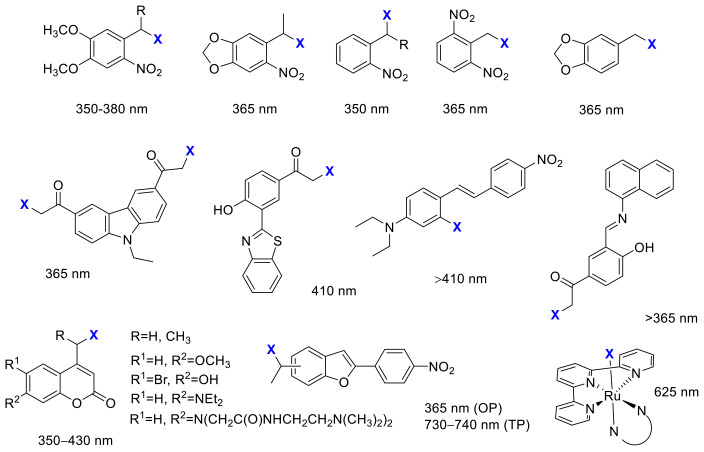
PPGs used in the discussed studies with their respective activation wavelengths.

The first application of photoremovable protecting groups (PPGs) as experimental tools for biological studies dates back to the 1970s. The photocage terminology coined at the advent of the field might be now ambiguous; therefore, nowadays, the photoremovable/photolabile/photoactivatable protecting group is commonly used in the literature. The design rationale behind PPGs is to mask the biological activity of a given substrate by covalently binding the protecting group to a moiety critical for the action (“caging”). The activity can be restored on demand in a spatiotemporally controlled manner by removing the PPG with light irradiation, i.e., the absorbed energy is translated into a photocleavage reaction (“uncaging”) [23]. The activation is irreversible, which is often considered as a disadvantage of the approach, as the released agents might still lead to unwanted effects upon diffusion or excretion. On the other hand, several PPG families of different properties are available, and temporary deactivation of the parent drug by adding a PPG is typically more straightforward due to the often significant structural differences upon caging. Moreover, with appropriate designs, dual or sequential, the wavelength-selective release of different agents could be envisaged as well (“chromatic orthogonality”) [24,25,26,27,28]. The PPGs used in the discussed studies with the respective uncaging wavelengths are summarized in Figure 2.

Initially, PPGs have found widespread application as experimental tools for studying dynamic processes, particularly in the field of neurophysiology [29,30]. Moreover, the number of studies on PPG-based light-activatable prodrug designs is steadily growing [31]. An optimal PPG-substrate construct should comply with several criteria already for in vitro applications, such as proper solubility, stability of the molecule to be released by light irradiation, efficient masking of the activity, clean and rapid photorelease, and cellular tolerance to the products of the photoreaction and the applied irradiation (dose-intensity, wavelength). The typical workflow for the rational design and evaluation of photoactivatable antitumor agents consists of the following steps: (i) determining the key pharmacophore for introducing the PPG unit, so that either the key ligand-target interactions are disturbed, or spatial conflicts arise rendering the caged drug inactive, (ii) assessing the photostability of the parent drug, so that its activity remains intact upon the light irradiation used for the uncaging step, (iii) assessing the hydrolytic stability of the caged prodrug at physiological pH, so that the parent drug is released exclusively upon light irradiation, (iv) determining the pharmacological activity of the parent and the caged drug molecule, assessment of the efficiency of masking (e.g., target enzyme inhibition, cellular assay), (v) studying the photolysis of the caged molecule, determining the conditions necessary for uncaging (wavelength and intensity of light irradiation, irradiation time necessary for complete conversion), (vi) determining the pharmacological activity of the caged molecule in the absence of light and following light irradiation, assessment of the efficiency of restoring the pharmacological activity, and (vii) verifying the tolerance of the experimental system to the light irradiation applied. Novel probes are typically characterized by UV/VIS absorption, hydrolytic stability, photocleavage rates, and quantum yields. Despite the complexity of the task [32], there is rapid development, and novel results on caged anticancer agents are discussed in the following sections.

### 2.1. Kinase Inhibitors

**Figure 3:**
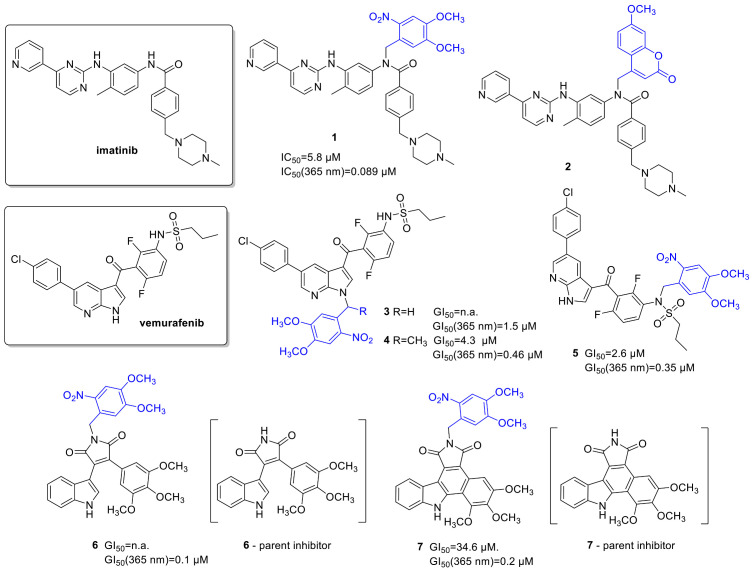
Photocaged kinase inhibitors, the PPG indicated in blue.

Kinase inhibitors affecting cell signaling pathways implicated in oncogenesis offer targeted therapy against specific malignant diseases. Introduced into therapy in the 2000s (2001—*imatinib*), the number of approved small-molecule kinase inhibitors was steadily growing. Although kinase inhibitors are the flagship of targeted therapy, their application is not without its challenges (e.g., development of resistance); therefore, they are a prime choice for further developments. The design of photoactivatable protein kinase inhibitors has been extensively studied by Peifer and coworkers (Figure 3). They designed a photoactivatable prodrug for *imatinib* [33], the Bcr-Abl tyrosine kinase inhibitor, which was approved for treating Philadelphia chromosome-positive chronic myelogenous leukemia, but started to be also used for other malignancies. Docking studies of *imatinib* in the ATP binding pocket of PDGF-Rβ showed two potential positions for attaching a PPG group to mask bioactivity: the NH in the benzanilide and in the *N*-arylpyrimidine-2-amine moieties, of which the first design was pursued in the study. As PPG, two groups were tested: a 4,5-dimethoxy-2-nitrobenzyl (DMNB) (**1**) and a coumarylmethyl scaffold (**2**); however, only the former provided efficient photorelease of *imatinib* in vitro at 365 nm (1 mM DMSO solution, 5.4 W, 10 min). In an enzymatic PDGF-Rβ assay, a 100-fold difference was detected between the parent *imatinib* (IC_50_ = 0.059 µM) and its caged prodrug (IC_50_ = 5.8 µM). The residual activity was assumed to originate from uncaged *imatinib* contaminating the sample. Following 365 nm irradiation, the bioactivity of *imatinib* could be nearly totally restored in vitro in the PDGF-Rβ assay, experimentally confirming a successful caging approach, while further bioactivity studies were not reported.

*Vemurafenib* (a BRAF^V600E^ serine/threonine kinase inhibitor) was approved for late-stage melanoma in 2011. Besides its therapeutic importance, *vemurafenib* tolerates the 365 nm UV irradiation relatively well, and chemical modifications could be straightforwardly carried out on its structure; therefore, it is an ideal candidate for the photoactivatable prodrug approach [34]. Photocaging would allow reaching higher target concentrations and thus avoid typical side effects, a high incidence of arthralgia (joint pain), skin rash, and the development of squamous cell carcinoma, which appear with higher dosage. Key pharmacophoric moieties for efficiently masking the activity of *vemurafenib* were determined by docking studies. In the subsequent work, two potential NH protection sites were assessed using *o*-nitrobenzylic PPGs (DMNB and 4,5-dimethoxy-2-nitrophenylethyl (DMNPE)), protection on the azaindole (**3**,**4**), and protection on the sulfonamide moiety (**5**) of *vemurafenib*. The former was expected to diminish hinge binding and therefore diminish activity on other kinases as well, whereas the latter was expected to allow residual activity on further kinases. As *N*-heterocycles are rarely described leaving groups, the authors studied the minimal structural requirements for the photoreaction as well, irradiating caged fragments of the parent compound besides the fully armed prodrugs (365 nm LED reactor, 5.4 W, 10 μM solutions in PBS buffer containing 10% DMSO, >90% conversion in 1 min). Regarding the photoreaction (i.e., rapid and quantitative photorelease), both protection sites proved to be feasible.

Next, the BRAF^V600E^ binding activity of *vemurafenib* and its caged prodrugs was determined, confirming a lower binding affinity for the caged inhibitors (K_d_: *vemurafenib*—10 nM, **3**—440 nM, **4**—77 nM, **5**—79 nM, overall ~8–40-fold difference). The selectivity profile was determined on a panel of 140 kinases, and in line with the modeling results, the azaindole-protected derivative **3** proved to be the most selective (activity on 2 kinases, vs. 13 for the sulfonamide-protected derivative **5** and 32 for *vemurafenib*). Antiproliferative activities were assessed in cellular growth assays (SKMel13, SKMel28, M14, and UACC62 melanoma cell lines), the caged compounds showing cytostatic activity at higher concentrations vs. the nanomolar cytotoxicity of the parent *vemurafenib* (GI_50_(SKMel13): *vemurafenib*—0.17 µM, **3**—no cell growth inhibition, **4**—4.3 µM, **5**—2.6 µM,). Irradiating the cells (SKMel13, 1.8 W, 5 min) at 365 nm after compound incubation, the cytotoxic activity of *vemurafenib* could be restored, although not totally, presumably due to incomplete photorelease (GI_50_: *vemurafenib*—0.19 μM, **3**—1.5 μM, **4**—0.46 μM, **5**—0.35 μM). In control experiments using Boc-Ala and Boc-Ala-DMNB model compounds, also the cleaved PPG itself was found to have antiproliferative activity, however at much higher concentrations than *vemurafenib* (GI_50_ = 34 μM). As further proof of restoring *vemurafenib* activity, the effect of caged molecules on ERK phosphorylation (SKMel13 cells) following UV irradiation was studied by Western blot analysis (complete suppression of ERK phosphorylation above 0.1 μM concentrations, dose dependency correlating with that of *vemurafenib*).

The same group also reported photoactivatable caged prodrugs for 3,4-diarylmaleimide antiangiogenic VEGFR-2 kinase inhibitors [35,36]. Based on the analysis of key ligand-target interactions by molecular docking, the hinge binder region was masked by a PPG unit (DMNB), potentially rendering the molecules inactive also toward other protein kinases. Docking of the caged prodrugs verified that protection disrupts key H-bond interactions to the hinge region as well as leads to steric hindrance hampering the access to the binding pocket. Uncaging was studied with 365 nm irradiation, confirming efficient photolysis of **7** (365 nm LED reactor, 5.4 W, 1 mM solution in DMSO, >90% conversion in 5 min) while the irradiation of compound **6** leads to a 1,6-π-electrocyclization not followed by oxidation to carbazole, making this derivative a less suitable candidate for further studies. VEGFR-2 kinase inhibitory assays confirmed a significant difference (300-fold) in the activity of the caged and the parent compounds. The same tendency was also observed in the in vitro antiproliferative assays (VEGFR-2 dependent PC-3 cells); however, in both studies, the caged inhibitors still showed some activity, ascribed to the presence of residual non-caged parent compound (GI_50_: **6**—not reached (parent drug—6.4 µM); **7**—35 µM (parent drug—0.2 µM)). Dose-response curves were recorded for both the parent and the caged compounds before and after irradiation (365 nm, 110 mW/cm^2^, 5 min). The activity of the parent drug was restored for compound **7**, whereas interestingly an increased activity was observed for compound **6** (GI_50_ after irradiation: **6**—0.1 µM (parent drug—2.9 µM); **7**—0.2 µM (parent drug—0.4 µM)). This was presumed to result from several factors: restored activity of the kinase inhibitor, the activity of the cyclized intermediate formed upon irradiation, the antiproliferative effect of the cleaved cage itself (confirmed by control experiments with Boc-Ala and DMNB-Boc-Ala model compounds, DMNB-Boc-Ala: GI_50_ = 50 µM) and a synergistic effect of the UV irradiation. Although tolerable in the studied case, the pharmacological effect or toxicity of the PPG itself might be a serious concern in therapeutic applications. On the positive side, caging did not hamper the cellular uptake of the inhibitors (studied on live PC-3 cells by fluorescence microscopy).

While looking at the photocaged kinase inhibitors, it is also worth noticing that several kinase inhibitors intrinsically possess interesting photochemical properties. Phototoxicity was reported for *vemurafenib* [37], while *dabrafenib* can be photodegraded to a novel fluorescent BRAF^V600E^ inhibitor [38].

### 2.2. Anthracyclines

**Figure 4:**
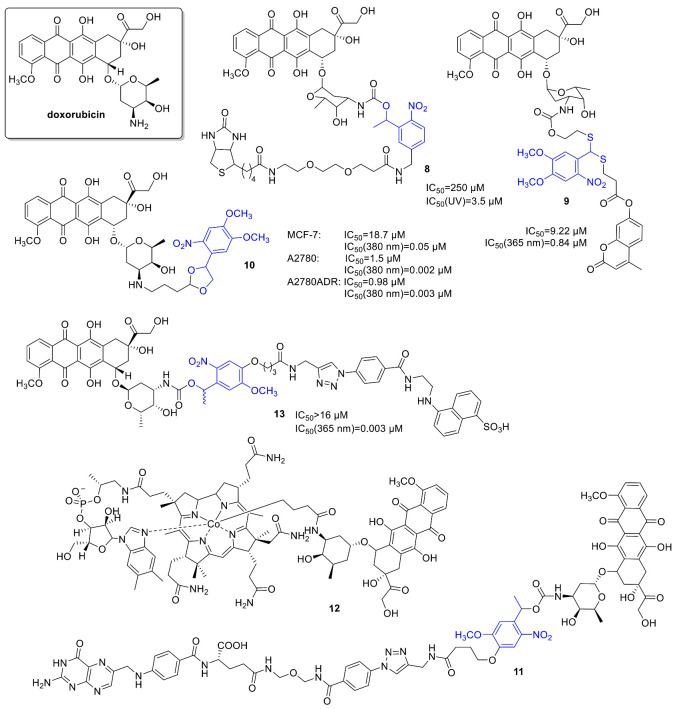
Photocaged *doxorubicin* derivatives, the PPG indicated in blue.

Anthracyclines have broad use in cancer chemotherapy, with a wide activity spectrum (e.g., solid tumors, hematological malignancies, soft tissue sarcomas). Regarding their activity, intercalation with DNA, affecting DNA replication and transcription and interaction with topoisomerase II have been studied. However, a very effective anticancer agent, the therapeutic use of *doxorubicin* is limited notably by its cardiotoxicity. For constructing a photoactivatable prodrug, Esener and coworkers bound a PPG unit (an *o*-nitrophenyl group) to an active amine on the sugar moiety of *doxorubicin*, a modification already demonstrated to decrease its toxicity [39]. Additionally, the construct was armed with a biotin group, linked via a water-soluble glycol spacer, for enhanced membrane interaction (**8**) (Figure 4). Biotin was intended to increase the clearance rate of the PPG-drug-construct and as well PPG fragment from the circulating blood using clearing agents after UV exposure. First, the release and release kinetics of *doxorubicin* in vitro upon UV irradiation was measured by LC-MS and NMR studies (in DMSO/water 1/9, 350 nm, 120 or 240 s of 2.19 mW/cm^2^ irradiation; kinetics: in DMSO/water 2/8, 350 nm, 0 to 10 min, 1.8 mW/cm^2^ irradiation: 1.8 µM/min *doxorubicin* release). The uptake and intracellular localization of the PPG construct vs. free *doxorubicin* (in 5 vs. 50 µM concentration) was studied in live PTK2 cells, using the intrinsic red fluorescence of *doxorubicin* (free or photocaged). Unlike the parent drug, the PPG construct was not entering the nucleus before light exposure, however following UV irradiation (60 s), the released free *doxorubicin* behaved similarly to the parent drug. Cytotoxicity was measured on A549 human lung cancer cells, showing a significant difference between the parent and the photocaged drug (*doxorubicin*: IC_50_ = 1.2 µM, doxorubicin-PPG construct: IC_50_ = 250 µM); however, the activity could be efficiently restored by UV irradiation in a light dose-dependent manner (samples were UV-irradiated in the media before incubation of the cells, i.e., cells were not irradiated directly. Maximal effect at 60 min: IC_50_ = 3.5 µM). To assess the feasibility of future applications, in a control experiment, A549 cells were irradiated (1.8 mW/cm^2^) but retained cell viability after 20 min of irradiation (after 60 min, 90% cell viability was recorded). To ascertain that drug release occurs only upon UV irradiation, the metabolic stability of the *doxorubicin*-PPG construct was verified using human liver microsomes. One major metabolite was identified, however, in small quantities, and its effect was not studied. Importantly, further in vivo studies were conducted [40]. First, the penetration of 365 nm light through ex vivo tumor tissue (irradiation from the center, penetrating light intensity measured at different angles) and the release of *doxorubicin* was measured using a LED/fiber-optic system (efficient release after 30 min irradiation). For conducting in vivo experiments, the *doxorubicin*-PPG construct was solubilized using Captisol^®^ cyclodextrin to allow a concentration of 1.2 mg in 200 μL of saline. The circulation was studied in vivo (female nude mice, alpha phase circulation half-life 10 min vs. ~20 min for *doxorubicin*). Of note, no free *doxorubicin* was detected neither in the serum nor in the urine out to 24 h, ascertaining UV irradiation as the major activation mode in subsequent experiments. In vivo drug release was studied on athymic nu/nu nude mice. A total of 10 min after injecting the *doxorubicin*-PPG construct (time point chosen based on the circulation half-life), a 365 nm LED was inserted into the middle of the tumor and turned on (240 μW/cm^2^, 30 min irradiation). A control tumor was left unirradiated, and blood samples were taken to monitor circulating drug concentration, whereas tumor samples were collected to analyze the distribution of the construct and the free drug. The construct was found in all tumor tissue sections of both the irradiated and unirradiated tumors, whereas a trace amount of *doxorubicin* was found in some sections of the unirradiated tumors vs. an increased *doxorubicin* content at the irradiation site (~6-fold difference in *doxorubicin*/gram of tumor tissue between irradiated and unirradiated tumors). The former was ascribed to the effect of UV leakage. No *doxorubicin* was detected in the serum sample taken after irradiation, pointing toward a controllable systemic exposure.

Choi and coworkers studied the design and synthesis of a novel thioacetal *ortho*-nitrobenzaldehyde (TNB) dual-arm photocage [41] and its application for constructing dual drug-fluorescent reporter conjugates for controlled release and real-time fluorescent monitoring of drug activation in parallel. Optimally the two payloads are released simultaneously by cleaving the linker C-S bonds (resistant to many chemical conditions), and several combinations of targeting ligands, drugs, etc., might be envisaged. For one set of drug-TNB-fluorescent reporter conjugates, *doxorubicin* was used as model anticancer substrate (*doxorubicin*-TNB-coumarin (**9**) (carbamate linkage)). The cleavage mechanism of the free cage was studied at 365 nm by ^1^H NMR, UV/VIS, and UPLC monitoring. A total of 365 nm photolysis of the cage conjugate in water/MeOH 1/1 led to an efficient release of the drug and reporter molecules presumably via a self-immolative mechanism (decay half-life (t_1/2_) < 2 min, Φ_uncaging_ = 0.08), the latter leading to an irradiation-dependent increase in fluorescence (i.e., the reporter is in a fluorescently quenched state before cleavage). The construct showed irradiation-dependent cytotoxicity in FAR(+) KB cells (before irradiation **9**: IC_50_ = 9.2 µM; after irradiation **9**: IC_50_ = 0.84 vs. *doxorubicin*: IC_50_ = 0.04 µM).

Hartman and coworkers studied the photocaging of 2-pyrrolino doxorubicin (2P-Dox) (**10**), a derivative of the standard *doxorubicin*, starting from its known diacetoxy prodrug [42]. Photorelease upon UV irradiation showed 88% release after 60 min irradiation, which corroborated by MS and HPLC results (20 mM in PBS, 380 nm, 9.0 mW/cm^2^). Cellular viability assays (CellTiter-Blue method) were run in three human cancer cell lines: MCF-7 (breast), A2780 (ovarian), and A2780ADR (*doxorubicin*-resistant ovarian), demonstrating a significant increase (327–750-fold) in activity upon light irradiation (380 nm, 30 min), activities compared with the parent drug. As a negative control, the released PPG itself was not cytotoxic up to 50 µM. Cellular uptake in MCF-7 cells was studied by flow cytometry, exploiting the fluorescence of *doxorubicin* and compound **10**. Compound **10** showed a higher cellular uptake than *doxorubicin*, in line with previous observations regarding the effect of alkylation with lipophilic moieties. Confocal microscopic studies indicated a nuclear co-localization of compound **10** and *doxorubicin*.

Controlled photorelease of the drug molecules could be combined with cell targeting to increase selectivity. Folic acid conjugates are often used to target overexpressed folate receptors (FRα) on tumor cells [43]. To release the internalized drug and avoid endosomal entrapment, Hartman and coworkers designed a photoactivatable folic acid-drug conjugate [44]. The conjugate consisted of folic acid, a PEG linker, and photocaged doxorubicin (**11**), using a DMNB unit as PPG (Figure 4). UV photolysis of the conjugate resulted in rapid release of *doxorubicin* (HPLC monitoring); however, no further biological assays were pursued.

Of further targeted carrier approaches beyond small molecules, Choi et al. described polyamidoamine (PAMAM) dendrimer nanoconjugates with folic acid ligands armed with a photocaged doxorubicin (using an *ortho*-nitrobenzyl based PPG). In vitro, on folic-acid-receptor-over-expressing KB cells, the doxorubicin constructs inhibited cell growth in an irradiation dose-dependent manner (but not without irradiation). The maximum effect observed (30 min irradiation, ~80% cell growth reduction) was comparable to the effect of the parent drug (~85% cell growth inhibition) [45].

For therapeutic applications, using appropriate (longer) wavelengths has several practical consequences. One approach to harness long wavelengths, consequently low-energy photons, is to target sufficiently weak chemical bonds that could be directly cleaved with lower energy. Lawrence and coworkers aimed to exploit the photohomolysis of the C-Co bond (<30 kcal/mol) in alkyl-cob(III)-alamins. The bond is weak enough to be cleaved by any wavelengths between 330 and 560 nm absorbed by the corrin ring system [46]. To move further toward the optical window of tissues and to develop wavelength-tunable probes, constructs with additional fluorophores serving as light-capturing antennas were designed (TAMRA, SulfoCy5, Atto725, DyLight800, Alexa700, Bodipy650). The design rationale was that upon excitation beyond cobalamin absorption wavelengths, the antennas might transfer excited-state energy to the corrin ring (coenzyme B12), thus contributing to Co-C bond cleavage. The full conversion was observed upon irradiation at the corresponding wavelength (Xe flash-lamp, 546 nm, 25 min); moreover, the constructs released the fluorophores also at the longer wavelengths (646/730/780 nm). Photorelease could also be operated by linking the fluorophores to the ribose 5′-OH of alkylcobalamins. To assess the application of cobalamin conjugates as drug delivery systems, a cobalamin-*doxorubicin* conjugate was prepared (**12**) [47]. Cytotoxicity was studied in HeLa (cervical cancer) cells (MTT assay at 10 µM), following different illumination times (30, 45, and 75 s at 530 nm). The cobalamin-conjugate affected cell viability in an irradiation-dependent manner (neither the construct without light nor the light irradiation without the construct had an effect on cell viability), with similar efficacy at the end point as that of the parent *doxorubicin*.

Hartman and coworkers also envisaged modulating the cellular entry of a cytotoxic agent by a photocage approach (photocaged permeability), linking *doxorubicin* as the model drug to a polar sulfonic acid fluorophore (EDANS) via a photocleavable nitroveratryl PPG (**13**) [48]. Photolysis was evaluated in PBS by HPLC monitoring (365 nm, 9.0 mW/cm^2^), showing parent drug release. The effect on cellular entry was studied on JH-EsoAd1 cells (Barrett’s esophagus associated adenocarcinoma cell line) under dark and light-irradiated conditions with flow cytometry. An irradiation-dependent fluorescence enhancement was observed both with flow cytometry and confocal microscopic studies. Cell viability was assessed with MTT assay, showing a clear correlation between decreased survival and light irradiation (365 nm, 9.0 mW/cm^2^, 20 min) of the probes (Dox: IC_50_ = 1.0 µM, EDANS-DOX: no toxicity till 16 µM, EDANS-DOX-lit: IC_50_ = 1.6 µM).

### 2.3. Tubulin (Dis)Assembly Inhibitors, Microtubule-Targeting Agents

**Figure 5:**
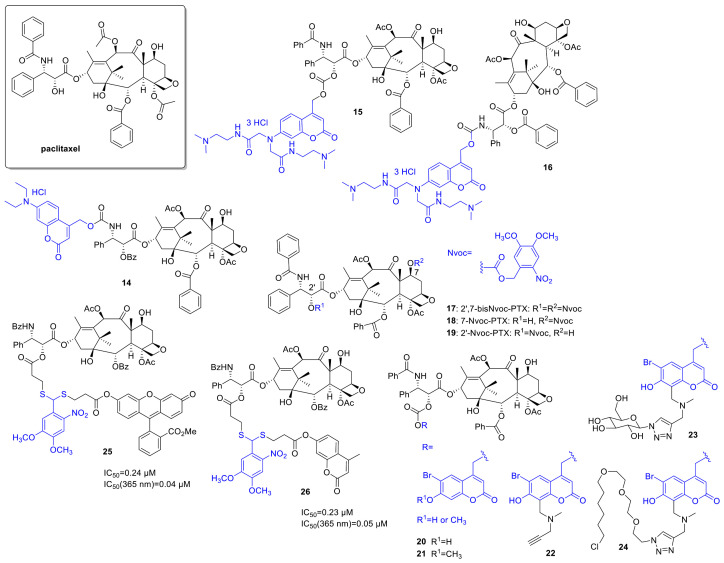
Photocaged *paclitaxel* derivatives, the PPG indicated in blue.

*Paclitaxel* (PTX) has a broad antitumor activity spectrum (e.g., breast, ovarian, head and neck cancers, malignancies resistant to conventional chemotherapy). Its action is based on affecting tubulin polymerization and microtubule dynamics, thereby disrupting cell division and interphase mechanisms and leading to cell death. Given the widespread therapeutic applications of PTX and the challenges limiting its use (e.g., resistance mechanisms, neuro-, and hematological toxicities), it is not surprising that several research groups proposed different photoactivatable prodrug designs. As one of the first examples, Kiso and coworkers linked a 7-*N*,*N*-diethylamino-4-hydroxymethyl coumarin (DECM) PPG to the *O*-acyl isoform of the parent drug (**14**) (Figure 5) [49]. Previous SAR studies suggested that the introduction will render the drug inactive (i.e., the structural modification is carried out at a key pharmacophore). Due to solubility reasons, the photolysis was studied in a 1/1 mixture of PBS and methanol at 430 nm (diode laser, 10 mW), monitored by UV/VIS absorption and HPLC (recovery rate: 69% after 30 min irradiation). The photorelease product subsequently yields the parent *paclitaxel* through O-N acyl migration at a reasonable rate under physiological conditions. To solve the solubility issue, a new coumarin PPG with improved water solubility was designed in a follow-up study by the same group [50] and linked to *paclitaxel* as carbonate (**15**), and 3′-*N*-debenzoylpaclitaxel (as carbamate, **16**) respectively (solubility over 100 mg/mL vs. 0.00025 mg/mL for *paclitaxel*). Despite the suitable water solubility, the carbonate prodrug was not stable enough under physiological conditions; therefore, it was not studied further (20% *paclitaxel* release after 8 h incubation at pH 7.4, 37 °C). In vitro photorelease from the carbamate, prodrug was studied in PBS, depending on the light source providing 24% (355 nm pulse laser, 10 Hz, 5 mJ, 4 min) or 69% (365 nm 6W UV-lamp) recovery rate of the parent *paclitaxel*. The lower recovery rate in the former case was ascribed to the sensitivity of the protecting group to intense light irradiation, besides the wavelength. No further biological assays were pursued, however.

Del Campo and coworkers used a 4,5-dimethoxy-2-nitrobenzyloxycarbonyl (Nvoc) PPG for preparing photocaged paclitaxels by blocking either one or both of two reactive hydroxyls (C2’, C7) critical for bioactivity (**17**, **18**, **19**) [51]. Photolysis of the caged probes was studied in ACN (5% water, 1 mM) solutions, with UV and HPLC monitoring (360 nm, 2.7 mW/cm^2^, chemical yields of PTX release: **17**—61%, **18**—89%, **19**—52%). In in vitro microtubule polymerization assay under dark and lit conditions, 7-Nvoc-PTX (**18**) showed residual activity, whereas 2′-Nvoc-PTX (**19**) and 2′,7-bisNvoc-PTX (**17**) operated only after UV exposure. However, for the double-protected drug, higher doses were necessary to obtain the same amount of released drug (according to HPLC studies, a sequential deprotection occurs). In cellular assay on HeLa cells, both monoprotected probes showed cytotoxicity, normal cell morphology was detected only following 2′,7-bisNvoc-PTX (**17**) treatment. Treatment with pre-irradiated probes led to similar results as observed with free PTX (i.e., the activity could be restored). Assessing cell viability in liquid HeLa cell culture, 2′,7-bisNvoc-PTX (**17**) was not cytotoxic till 10 µM, whereas 2′-(**19**) and 7-Nvoc-PTX (**18**) showed ~50% viability at 0.1 µM. A further demonstration of the need for double protection. Masking of activity and restoring it upon pre-irradiation was also confirmed in in vitro assays of microtubule catastrophes and mitotic index (HeLa cells). In situ photolysis by a short light pulse under a fluorescent microscope resulted as well in the reorganization of microtubules.

To broaden the structural diversity of PPGs, Furuta and coworkers designed a modular 6-bromo-7-hydroxycoumarin-4-ylmethyl (Bhc) group that has a terminal alkyne moiety allowing further functionalization via click chemistry and used it for the synthesis of caged PTXs [52]. Besides a Bhc cage-PTX conjugate (**20**, **21**), two further probes were prepared exploiting the alkyne chemical handle (**22**), one functionalized with a sugar to improve water solubility (**23**), and another with a halo tag ligand for cellular targeting (**24**). PTX was released from all novel probes under in vitro conditions at 350 nm (KMOPS containing 0.1% DMSO, 10 mJ/s lamp, Φ_dis_ = 3.5–14), the improved water solubility of compound **23** also translating to more efficient photolysis, presumably by promoting ion-pair intermediates implicated in the reaction. Masking and UV-triggered restoring of the activity of the parent drug was assessed in an in vitro tubulin polymerization assay (porcine brain tubulin, turbidity assay at 340 nm), confirming an irradiation-dependent effect (350 nm, 10 mJ/cm^2^, 60 s) and comparable maximum activity of activated caged compounds with *paclitaxel*; however, no animal studies were undertaken.

For the thioacetal *ortho*-nitrobenzaldehyde dual-arm photocages described in the previous section, PTX was used as another study drug (*paclitaxel*-TNB-fluorescein (**25**, ester linkage), *paclitaxel*-TNB-coumarin (**26**, ester linkage)). Efficient photorelease was also detected for the PTX-constructs (**25**/**26**: Φ_uncaging_ = 0.05/0.07). Cellular uptake and reactivation after uptake of **25** were studied by fluorescence with flow cytometry in FAR(+) KB cells. The results confirmed an intracellular accumulation of the PTX-TNB-fluorescein conjugate **25** and an efficient 365 nm photorelease of the fluorescent reporter intracellularly (365 nm, 2 min). The correlation between the cytotoxicity and fluorescence was studied in a microplate assay on FAR(+) KB cells (XTT assay). Dose-dependent cytotoxicity was recorded both for irradiated and non-irradiated conjugates; however, cytotoxicity prior irradiation was modest compared to the parent drug (before irradiation **25**/**26**: IC_50_ = 0.24/0.23 µM; after irradiation **25**/**26**: IC_50_ = 0.04/0.05 vs. PTX: IC_50_ = 0.02 µM). Moreover, the cytotoxicity was in correlation with the increase in fluorescence upon irradiation, paving the way toward quantification and effective real-time monitoring of the drug release process. Real-time fluorescence in cellular systems was recorded over extended periods of time following irradiation to study the release kinetics (24 h monitoring, 0.5 or 2 min irradiation). The gradual, continuous increase in fluorescence was ascribed to the conversion of transient dye intermediates to parent fluorescent probes. On the other hand, a certain degree of increase in fluorescence was observed for the conjugates even without UV irradiation after extended periods, i.e., a dark release was occurring.

Dobber et al. designed a photocaged small-molecule tubulin inhibitor starting from the previously described inhibitor CMPD1 and using DMNB as PPG [53], linked to a phenolic OH group, the crucial role of which has been confirmed by previous SAR studies. First, the UV-stability of the parent inhibitor was ascertained at 365 nm, followed by measuring the kinetics of the photorelease from the cage monitored by HPLC (2.7 W, τ = 0.61 min, maximum parent drug concentration reached after 2 min irradiation). For assessing the pharmacological effects, cell viability assays on U251 and patient-derived RN1 glioblastoma cells were used, after first checking their UV-irradiation tolerance (1.8 mW till 5 min, maximum tolerated light exposure: U251—1 min, RN1—30 s). A 55- and 125-fold difference was recorded between the activity of the parent and the caged drug without UV irradiation. UV irradiation of the cage-treated cells could recover almost totally the cytotoxicity (photocaged drug after irradiation—EC_50_(U251) = 2.1 μM, EC_50_(RN1) = 1.2 μM). Additionally, in control experiments blocking the pharmacophoric OH with a bulky aromatic group and cytotoxicity of the released DMNB were addressed (released DMNB did not change cell viability till 100 µM concentration in U251 cells). Loss of activity of the caged drug and recovering the activity upon UV irradiation were further addressed in in vitro tubulin polymerization (porcine brain tubulin) and tubulin binding assays (competitive colchicine binding assay), as well as via assessing the effect on the tubulin network and cell morphology in U251 glioblastoma cells (immunofluorescence staining of β-tubulin) and measuring the apoptotic effect on RN1 glioblastoma cells (Annexin V staining). All the pharmacological studies pointed toward the release of a bioactive molecule from the photocage upon UV exposure and inactivity without UV irradiation.

### 2.4. DNA Alkylating Agents

**Figure 6:**
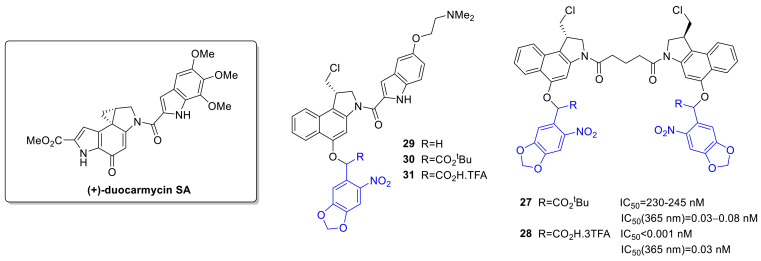
Photocaged duocarmycin derivatives, the PPG indicated in blue.

Alkylating agents cause direct DNA damage (consequent fragmentation, crosslinking or mutations due to mispairing) and thereby disrupt DNA replication and cell division. The nitrogen mustards were among the first anticancer chemotherapeutics, and alkylating agents have widespread use against a variety of hematological and solid tumors. Nitrogen mustards typically have a bis(2-chloroethyl) group important for action, e.g., also in *chlorambucil*. Schuberth et al. developed photoactivatable prodrugs for the highly cytotoxic duocarmycin [54]. Despite their cytotoxic potential (activity in multidrug-resistant cells), no duocarmycins are yet approved for therapy. (+)-Duocarmycin SA contains a DNA recognition and a DNA alkylating unit, the latter implicated in forming DNA adducts. In the cited study, a seco- and a dimeric duocarmycin analog was used as the parent drug, and the PPG (3,4-methylenedioxy-ONB—with an additional *tert*-butyl ester or carboxylic acid at α-position, overall 5 novel probes) was linked to a phenolic OH to hinder the formation of the spiro-cyclopropyl unit critical for action (Figure 6). Photorelease was studied in PBS buffer (1/10% DMSO, 365 nm, 1.1 mW/cm^2^) with LC-MS monitoring. For compounds **27**, **28,** the photoreactions proceeded via the expected pathway, i.e., PPG cleavage and subsequent cyclization (**28**: Φ = 0.45). For the **29**–**31** series, however, the photorelease was slow and led to the formation of several side products. Cellular cytotoxicity was assessed on A549 bronchial carcinoma cells (HTCFA-derived test) with pre-irradiated samples (365 nm, 1.1 mW/cm^2^, 30 min) or without UV exposure. For **27,** a more than 106-fold difference of activity was recorded vs. the parent drug, and the activity could be efficiently restored with UV exposure. Interestingly, for **28,** the PPG construct itself was found to have enhanced cytotoxicity vs. the free drug. No proper phototherapeutic index values were found for the **29**–**31** series.

**Figure 7:**
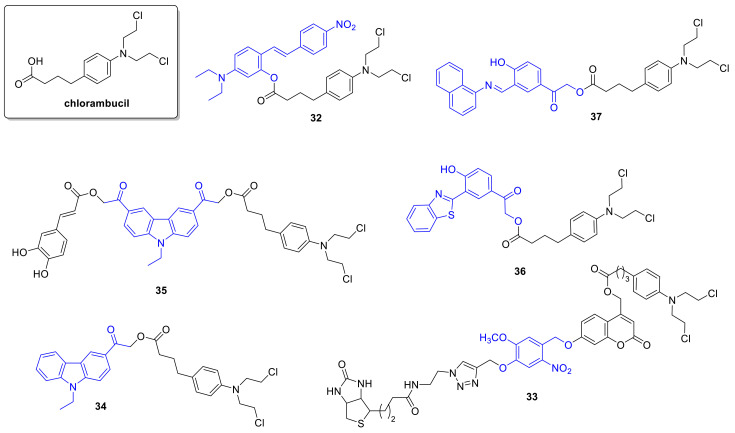
Photoactivatable *chlorambucil* derivatives, the PPG indicated in blue.

Several photoactivatable prodrug approaches were described for the aromatic nitrogen mustard *chlorambucil* (Figure 7), used for chronic lymphocytic leukemia, Hodgkin lymphoma, and non-Hodgkin lymphoma. Push-pull stilbenes were devised with the aim to develop visible light-activated PPGs with turn-on fluorescent real-time monitoring ability [55]. The PPG was based on a trans-4-(*N*,*N*-dimethylamino)-4′-nitrostilbene unit, placing the substrate (an alcohol or a carboxylic acid) to be liberated at the point of photocyclization, serving as a leaving group upon the process. By formation of the cyclized phenanthrene product, a blue fluorescence appears that was used for monitoring of the photorelease. Besides simple model substrates, as model drug molecules for photorelease studies, *chlorambucil* was selected (**32**). Due to the presence of appropriately placed electron-donor and –acceptor groups and consequent charge transfer, the novel caged derivatives had an absorption maximum typically above 400 nm, whereas the fluorescence became quenched in polar solvents. The uncaging was studied in ACN (medium-pressure mercury lamp (125 W)), with NMR, UV/VIS, HPLC, and MS monitoring with ≥410 nm irradiation, confirming an efficient liberation of the caged substrates accompanied by an increase in fluorescence (450 nm) (*chlorambucil*: Φ_p_ = 0.10). Modeling physiological conditions, uncaging of *chlorambucil* was also measured in ACN/HEPES (1/19) mixture (λ ≥ 410 nm, 75% release after 1 h, Φ_p_ = 0.02). Real-time monitoring of photorelease was studied in MCF-7 cells by confocal microscopy after different light exposure periods (λ ≥ 410 nm, 0–30 min). Irradiation-dependent cytotoxicity of caged *chlorambucil* was assessed on MCF-7 cells (MTT assay), confirming a significant drop of cell viability in the irradiated samples vs. the unlit ones (dark sample: more than 75% cell viability (100 µM), after 30 min irradiation: 15% viability, IC_50_ = 230 µM).

As a second approach, a photoactivatable double prodrug strategy was developed to increase the selectivity of drug release (“dually locked photoresponsive DDS”) [56]. *Chlorambucil* was linked to a first 7-hydroxycoumarin, that was itself masked with an *o*-nitrobenzyl PPG quenching its fluorescence (fluorescent quantum yields: coumarin-*chlorambucil* (CC)-Φ_f_ = 0.49, ONB-CC-Φ_f_ = 0.010). To provide tumor-targeting ability, a biotin ligand was used as well (**33**). Photolysis was studied with ^1^H NMR, HPLC, and fluorescence monitoring (10 µM, water/ACN 4/1, 125 W, ≥365 nm). The fluorescence (546 nm) increased within 5 min irradiation and reached a maximum in 30 min, confirming the turn-on ability of the construct. After 5 min irradiation, CC release (90%) and nitrosobenzaldehyde formation occurred. The second uncaging (*chlorambucil* release, 70%) necessitated a longer, 20 min irradiation (however, less than 7% release with diode laser, 730 nm, 30 mW/cm^2^). Incubating MDA-MB-231 (breast adenocarcinoma) cells with ONB-CC and subjecting them to a UV-irradiation regime, a gradually intensifying green fluorescence appeared, confirming intracellular accumulation and CC release. Cytotoxicity was studied on the MDA-MB-231 cell line with UV irradiation (30 min, ≥365 nm). At 50 µM concentration, a 50% cell viability was observed (vs. *chlorambucil* IC_50_ = 67 µM). Without light irradiation, no significant effect was detected for the drug-PPG construct. Higher apoptotic activity observed for the biotin-tagged construct in cell cycle analysis pointed toward a contribution of accumulation to the increased cytotoxicity.

The same group used PPGs for the design of dual drug delivery systems [57]. As a photoactivatable and fluorescent unit allowing imaging besides caging, an acetly-carbazole was used, from which single and dual-arm cages were prepared (**34**, **35**). In the latter case, either two of the same or two different substrates could be linked as esters. Photorelease was first studied in ACN/water 7/3 solutions at 365 nm using various acid model substrates, confirming a clean process (i.e., formation of the parent 3-(hydroxyacetyl) 9-ethyl 9*H* carbazole and the uncaged free acid substrate) and suitable recovery of the free acid (medium-pressure mercury lamp (125 W), Φ_p_ ~0.1). Importantly, dual-arm cages released two equivalents of the linked carboxylic acids, and a simultaneous release occurred in the case of dual-arm cages with two different acid substrates. For a proof-of-concept study of dual drug delivery systems, caffeic acid and *chlorambucil* were selected as the bioactive antitumor compounds. Photorelease of the parent drugs was verified in ACN/water 7/3 solution (medium-pressure mercury lamp (125 W), 0.1 mM, 60 min irradiation, caffeic acid: 91% release, Φ_p_ = 0.046; *chlorambucil*: 94%, Φ_p_ = 0.051). The cellular uptake of the dual-arm drug-PPG conjugate was studied in glial cancer cells (U87MG) by confocal fluorescence at 10 µM concentration (3 h treatment), confirming an internalization of the conjugate. The in vitro cytotoxicity of the conjugate without and after irradiation was determined on normal HaCaT cells, confirming feasible biocompatibility (80% cell viability before irradiation, 40% cell viability after irradiation at 80 µM concentration). The anticancer activity of single-arm PPG-caffeic acid, PPG-*chlorambucil,* and the dual-arm PPG-conjugate was studied on U87MG cells by MTT assay. A significant difference in cell viability was recorded between the experiments without and after UV irradiation (more than 70% cell viability for all constructs at 25 µM concentration, after irradiation: PPG-caffeic acid: IC_50_ = 15 µM, PPG-*chlorambucil* **34**: IC_50_ = 15 µM, PPG-caffeic acid-*chlorambucil* **35**: IC_50_ = 9 µM).

Further development led to the modifications of the *p*-hydroxyphenacyl PPG to render it fluorescent and activatable above 400 nm [58]. A 2-(2’-hydroxyphenyl)benzothiazole unit was added, known to have a fast and efficient excited-state intramolecular proton transfer (ESIPT) effect, contributing to interesting fluorescent properties potentially allowing real-time monitoring of photorelease processes. The novel PPG system was tested for caging *chlorambucil* (**36**). In vitro photorelease was assessed in ACN/HEPES 1/19 (0.1 mM, 410 nm, medium-pressure mercury lamp (125W), Φ_u_ = 0.46, 90% release after 15 min irradiation). The ESIPT-assisted photorelease mechanism was further addressed with computational chemistry, supporting a singlet-state ESIPT followed by intersystem crossing (ISC) and triplet-state photo-Favorskii rearrangement. The in vitro photoreaction was accompanied by a fluorescent color change from green (517 nm) to blue (450 nm). In the cellular environment (MDA-MB-231 cells), after internalization of the PPG-*chlorambucil* constructs, the photorelease could be followed by confocal microscopy (≥410 nm, 15 min irradiation). Cytotoxicity of the parent and caged drug was assessed on MDA-MB-231 cells with MTT assay. Without irradiation, the cell viability remained above 90% for different PPG-*chlorambucil* concentrations; however, ≥410 nm irradiation efficiently restored the activity of the parent drug. To address tissue penetration issues, a *p*-hydroxyphenacyl-based two-photon (TP) responsive system for caging *chlorambucil* [59] was also designed. The novel PPG system showed ESIPT and aggregation-induced emission (AIE) phenomenon. Upon in vitro photolysis (ACN/PBS buffer) of the PPG-*chlorambucil* conjugate (**37**) (≥365 nm), a fluorescence color change was observed (550→430 nm blue shift), potentially allowing real-time monitoring of the photorelease (0.1 mM, ACN/PBS 1/9, medium-pressure mercury lamp (125 W), ≥365 nm, 15 min, Φ_u_ = 0.49). Drug release could be effectuated sequentially as well with an on-off irradiation regime, thus verifying the light-dependency of the process. Uncaging occurred from the aggregate state, as confirmed with irradiation of the conjugate in the presence of different water fractions. Two-photon photorelease was confirmed experimentally at 700 nm (0.1 mM in DMSO/water 1/9, 25% release after 3 h) with an open aperture Z-scan technique with a pulsed laser. In vitro cytotoxicity studies were conducted in MCF-7 (breast cancer) cells, confirming first the cellular uptake of the conjugate. Upon 15 min ≥ 365 nm irradiation, the fluorescent color change due to photorelease could be monitored by confocal microscopy, and a concentration- and irradiation-dependent cytotoxic effect was recorded using the MTT assay (cell viability above 95% before irradiation, after irradiation IC_50_ = 31 µM).

### 2.5. PARP Inhibitors

**Figure 8:**
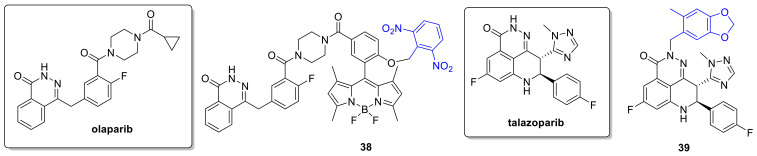
Photoactivatable PARP inhibitors, the PPG indicated in blue.

Poly (ADP-ribose) polymerase (PARP) inhibitors target DNA damage response, particularly in cancer cells with high replication stress and genomic instability. The first in class *olaparib* was approved in 2014, *talazoparib* in 2018. Indications include BRCA mutated ovarian and breast cancer, and clinical trials addressing further indications and combination therapies were launched. Weissleder and coworkers aimed to develop a construct allowing real-time monitoring of intracellular drug transport using a photocaging concept [60]. As fluorophore, a BODIPY unit was selected, a 2,6-dinitrobenzyl (DNB) as PPG and as the study drug a derivative based on the PARP inhibitor *olaparib* (**38**) (Figure 8). PPG-binding quenched fluorescence that was restored upon 365 nm irradiation (1.6 mW/cm^2^, ~45 min). In an in vitro PARP1 activity assay, both the caged and released derivatives had activity in the nanomolar range (**38**: IC_50_ = 32 nM, **38**-uncaged: IC_50_ = 68 nM). Cytotoxicity was measured on MDA-MB-436 cells, showing higher cytotoxicity for the conjugates than the parent drug (presumably due to increased cellular retention). Real-time monitoring of cellular drug efflux at single-cell resolution at steady-state conditions was demonstrated after photoactivation (405 nm laser).

Zhou and coworkers studied photocaging of *talazoparib*, an approved oral nanomolar PARP inhibitor drug [61]. The key pharmacophore for introducing the PPG unit (the lactam moiety) was determined by molecular docking so that masking disrupts hydrogen bonding interactions with the protein besides steric clashes to render the caged inhibitor inactive (**39**). As PPG, the *o*-nitrobenzyl unit was selected, allowing fast and complete release of *talazoparib* upon 365 nm irradiation (20 µM in methanol, 4.1 mW/cm^2^, 5 min). Recording the dose-response curves of *talazoparib* and its caged counterpart on PARP-1 enzyme, a 380-fold difference was found (*talazoparib*: IC_50_ = 0.005 µM, photocaged drug: IC_50_ = 1.9 µM). Further assessment of the pharmacological effects at a cellular level (inhibition of poly ADP-ribosylation in HeLa cells) also showed a significant, 658-fold difference between the activity of the free and the caged drug molecules. Cytotoxicity of the parent and caged *talazoparib* was studied in BRCA1- and BRCA2-defective cell lines (MX-1, Capan-1: *talazoparib*: IC_50_ = 0.015 µM and 0.003 µM; photocaged drug: IC_50_ = 1.9 µM and 0.86 µM), confirming the former tendencies (i.e., the caged version being significantly less active). For testing whether the pharmacological activity could be restored, the irradiation times were a trade-off between the photolysis conversion and the UV-tolerance of the cell lines (MX-1/Capan-1—1/3 min). This could be the reason for not obtaining the maximal pharmacological effect of the parent compound following UV irradiation (MX-1: IC_50_ = 0.58 µM, Capan-1: IC_50_ = 0.092 µM); however, an increased effect was recorded compared to the caged molecules (3- and 9-fold difference vs. the non-irradiated group). On the other hand, the released PPG was not found to have cytotoxic effects in the studied concentrations (using a Boc-Ala-PPG model); therefore, the observed pharmacological activity could be fully ascribed to the released *talazoparib*.

### 2.6. Histone Deacetylase Inhibitors

**Figure 9:**
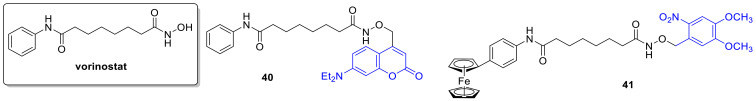
Photoactivatable HDAC inhibitors, the PPG indicated in blue.

Epigenetic modulators are an important and large family of anticancer agents, therefore a logical choice of great potential for designing photoactivatable agents. Epigenetic processes have important regulatory functions, and histone acetylation affects DNA transcription and gene expression. The operation of histone deacetylases (HDAC) diminishes gene transcription and differentiation, and histone modification is implicated as well in cancer pathogenesis. The first in class pan-inhibitor (class I and II HDACs inhibitor) suberoylanilide hydroxamic acid (*vorinostat*, SAHA) was approved in 2006 for cutaneous T cell lymphoma. Nakagawa and coworkers studied photocaging of SAHA [62] used in clinics (**40**) (Figure 9). The key pharmacophore moiety of HDAC inhibitors is a zinc-binding group, a hydroxamate in the case of SAHA. Therefore binding the PPG, a (7-diethylaminocoumarin-4-yl)methyl group was planned at this site to block activity. Photorelease was studied in Tris-DMSO (20%) at 400–430 nm with HPLC monitoring (66 mW/cm^2^; photodecomposition Φ = 4.5 × 10^−3^, SAHA release Φ = 1.5 × 10^−3^), leading beside the parent inhibitor and the PPG to the formation of partly unidentified side products as well. The caged drug showed an irradiation-dependent HDAC inhibition (HDAC-Glo I/II assay kit), whereas the main photoproducts besides the parent drug (7-diethylamino-4-hydroxymethylcoumarin, suberanilic acid) were ineffective (at 0.5 µM). Cell growth inhibition studies were conducted on HCT116 cells, confirming a cage concentration- and light exposure-dependent effect (1, 3, or 10 min irradiations).

Gasser and coworkers chose a light-stable ferrocene derivative of SAHA (Fc-SAHA) as a starting point for their studies and masked it with a DMNB moiety at the hydroxamic acid (**41**) [63]. Photorelease kinetics and profile were studied using a UV reactor (300–400 nm, centered at 350 nm) with LC-MS monitoring (10 min irradiation, 2.79 J/cm^2^). Besides the parent drug released, an amide analog of Fc-SAHA was detected, and further minor unidentified by-products. In vitro HDAC inhibition was tested under dark and irradiated conditions (10 min, 2.79 J/cm^2^—dose selected as a trade-off between conversion and UV-tolerability), confirming an efficient masking (26- to 333-fold) and light-triggered release of the activity on HDAC1, HDAC2, and HDAC6. The remaining effect of the caged drug might be a result of insufficient steric bulk of the applied PPG. No further cellular assays were performed due to insufficient aqueous solubility of the caged drug.

### 2.7. Metal Complexes

**Figure 10:**
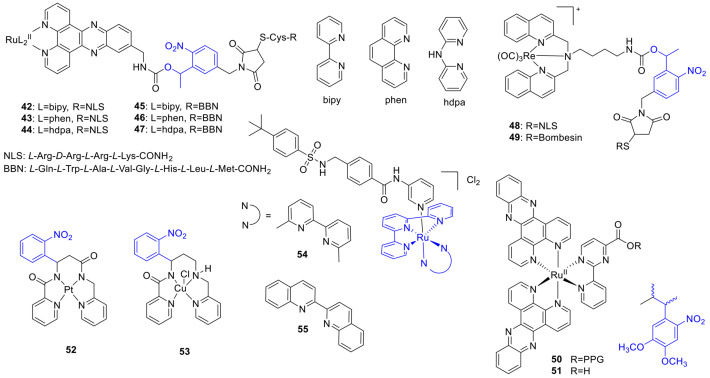
Photoactivatable metal complexes, the PPG indicated in blue.

The use of metal-based drugs in cancer therapy has been limited by the challenges of distinguishing between therapeutic and toxic doses. *Cisplatin* was approved in the 1970s and is effective against a plethora of cancers. Its activity is based on crosslinking DNA purine bases, affecting DNA repair processes, leading thereby to DNA damage and subsequent cell death. Photoactivation in the context of inorganic chemistry started to appear in the 1990s. Photoactivated chemotherapeutics (PACT) describe approaches wherein upon light activation, a structural change and consequently a change in bioactivity occurs. PACT was extended to several metal complexes. The photoactivation could occur via different mechanisms, as photoreduction, photosubstitution, or C-C bond cleavage. In the following sections, illustrative examples based on conventional photocage or photoswitch motifs are presented. For further information on photoactivatable metal complexes, we refer to the respective literature [32,64,65]. Mari et al. designed Ru^II^–polypyridyl complexes combining a tumor-targeting (a bombesin—BBN or a nuclear localization signal sequence—NLS) and a light-activatable functionality (an *o*-nitrobenzyl PPG) to achieve double selectivity [66]. Three complexes with different ligands conjugated to the PPG and NLS and three conjugated to the PPG and BBN and their negative controls without the light-responsive unit were prepared (**42***–***47**) (Figure 10). A total of 350 nm irradiation in PBS efficiently released the parent Ru^II^ complexes from the cages within 6–10 min (1.6–2.6 J/cm^2^, photorelease quantum yields: 0.4–2.1%), whereas upon irradiation in ACN no ^1^O_2_ production was observed for any of the probes. Cytotoxicity (fluorometric cell viability assay) and cellular localization (luminescence microscopy) studies were run on normal MRC-5 (fetal lung fibroblast) and cancerous HeLa cells, either keeping the cells in the dark following incubation or irradiating them at 350 nm (10 min, 2.6 J/cm^2^). Under dark conditions, the free Ru^II^ complexes showed no cytotoxicity (IC_50_ > 100 µM); however, two of them showed modest cytotoxicity (IC_50_ = 65–83 µM) upon light irradiation on HeLa and MRC-5. Two of the fully armed NLS constructs (**42**, **43**) showed an accumulation in the nucleus, two NLS constructs showing dark toxicity as well (not increased by UV irradiation), and overall hardly any selectivity was found for this design. The cellular localization of BBN-conjugates depended on the Ru^II^-ligands (nucleus/cytoplasm). Two of the BBN-conjugates showed a light-induced increase in toxicity on HeLa cells; however, the phototoxic index values were modest (max. 2-fold). The lack of cytotoxicity observed on MRC-5 cells could be the result of a more efficient uptake into cancerous cells resulting from the targeting peptide conjugation. Controls without the light-responsive unit showed no toxicity (neither NLS nor BBN controls). The combination of light activation and cellular targeting was also studied in the context of Re^I^ complexes (**48**, **49**) [67].

Gasser and coworkers extended photocaging onto a cytotoxic, coordinatively saturated and substitutionally inert Ru^II^ complex ([Ru(dppz)_2_(CppH)]^2+^ (**51**)), adding a 3-(4,5-dimethoxy-2-nitrophenyl)-2-butyl (DMNPB) PPG to a carboxylate necessary for its activity (**50**) [68]. The novel probe was sufficiently stable in PBS at 25 °C (7% **51** release over 24 h). UV photolysis (350 nm, in PBS) was studied with UV/VIS and UPLC-MS monitoring (20 min, 5.16 J/cm^2^, ≥92% release, 3.8% quantum yield for photorelease). Cellular cytotoxicity was assessed on HeLa, U2OS (bone cancer), and MRC-5 (non-cancerous lung fibroblast) cell lines under dark and light-irradiated conditions (resazurin fluorometric assay). The caged probe was not cytotoxic till 100 µM over 4 h, whereas light irradiation (350 nm, 10 min, 2.6 J/cm^2^) efficiently restored the activity (4 h, IC_50_ (µM), HeLa: **51**-dark = 16, **51**-UV = 6, **50**-UV = 17; U2OS: **51**-dark = 30, **51**-UV = 13, **50**-UV = 17; 48 h, IC_50_ (µM), MRC-5: **51** = 15, **50** = 85). As a negative control, neither light irradiation itself nor DMNPB had cytotoxic effects. Of note, **51** itself has photoinduced cytotoxicity contributing to the final effect due to singlet oxygen production.

Franz and coworkers developed a photocaged Pt^II^ complex, wherein the ligand backbone could be cleaved upon light irradiation (using a nitrophenyl PPG) to allow ligand exchange and subsequent toxicity ([Pt(cage)]) (**52**) [69]. Photolysis in pH 7.4 phosphate buffer monitored with UV/VIS spectroscopy showed a complete conversion in ~2 min (Φ = 0.75), and with LC-MS, the photoproduct could be identified as the result of two bond cleavages. Cytotoxicity was studied in MCF-7 cells in comparison with *cisplatin* (LDH release assay). Under dark conditions, cells remained viable following [Pt(cage)] treatment (up to 200 µM, over 96 h), whereas UV irradiation significantly increased the cytotoxicity of the probe. However, in control experiments, the ligand backbone itself was found to be cytotoxic, and this effect was tuned down upon Pt^II^-coordination. Regarding the mechanism of action, agarose gel electrophoresis did not confirm DNA interactions. The formation of a charged complex from a neutral one upon photolysis was postulated to contribute to cell accumulation.

Further development led to a photoactivatable Cu-complex with a photolabile nitrophenyl group (3GCage; formulated as [CuCl(3Gcage)], Cu3G) (**53**) [70,71]. UV irradiation releases the Cu^II^ by cleaving the multidentate chelator backbone (dissociation constant 0.18 fM at pH 7.4). Complexing the Cu with 3GCage inhibits Cu-induced hydroxyl radical formation, whereas, upon UV photolysis, a 300% increase in hydroxyl radical formation occurs. To assess whether the reducing cellular environment could lead to Cu^I^ release (in the absence of light), cyclic voltammetry in pH 7.4 PBS buffer was run, giving similar results as that of the stable Cu(Atsm) bisthiosemicarbazone complex. Further experiments with various reducing or binding agents in pH 7.4 PBS buffer provided additional indications of stability under most conditions. The cytotoxicity of Cu3G was evaluated in vitro in MCF-7, HeLa, and HL-60 cancer cells (CellTiter-Blue fluorometric assay). Little effect on cell viability was observed under dark conditions below 100 µM (IC_50_~150 µM), whereas following UV exposure (350 nm, 90 s, conditions sufficient for full conversion) led to a decreased viability in the different cell lines (IC_50_~75 µM). H_2_O_2_ (non-toxic dose of 50 µM) had a synergistic effect on cytotoxicity (dark: >80% viability up to 100 µM, UV: IC_50_~30 µM). UV irradiation of the 3GCage was well tolerated by the study system (up to 200 µM), the effect of extracellular copper or the UV irradiation was ruled out by control experiments. Microscopic studies showed extensive cytoplasmic vacuolization in light-irradiated Cu3G/Cu3G+H_2_O_2_-treated cells, a sign of non-apoptotic cell death. This pathway could be of interest for treating apoptosis-resistant cell lines.

Metal complexes could serve as caging groups to release organic substrates upon light irradiation. Bonnet and coworkers described ruthenium complexes ([Ru(tpy)(dmbpy)(L)]^2+^ and [Ru(tpy)(biq)-(L)]^2+^) caging a nicotinamide phosphoribosyltransferase (NAMPT) inhibitor (STF-31) (**54**, **55**) [72]. The pyridine moiety of the parent drug could coordinate to ruthenium and could be photodissociated in water (625 nm, photosubstitution quantum yields: **54**: Φ = 0.058 (rt), 0.080 (37 °C); **55**: Φ = 0.013 (rt), 0.019 (37 °C)). Cytotoxicity was tested on A549, MCF-7, A431, and non-cancerous MRC-5 cell lines under dark and light-activated (628 nm, 20.6 J/cm^2^) conditions (SRB assay). For **54** limited difference was recorded between dark and irradiated samples (phototherapeutic index 0.67–1.3), presumably due to thermal instability. More significant differences were found for complex **55**, with modest cytotoxicity against MRC-5 cells under dark conditions (EC_50_ = 46 µM) and increased activity on cancer cells upon irradiation (EC_50_ = 7.1–19 µM, phototherapeutic index 1.6–3.6). Given the modest singlet oxygen-producing efficiency of the probes, the effect was ascribed to the STF-31 released. Indeed, re-running the assays under hypoxic conditions (1% O_2_) provided similar results for **55**. Light activation was corroborated also with in vitro NAMPT inhibition assay (**55**-dark: IC_50_ = 4.8 µM, **55**-lit: IC_50_ = 0.26 µM, STF-31: IC_50_ = 0.25 µM).

### 2.8. Nitroxide Donors

Depending on the cellular microenvironment, nitric oxide (NO) could exert various biological functions. NO donors are being studied in the context of carcinogenesis and as potential anticancer agents. Given the various potential effects of NO, a localized action would be, however, of paramount interest. Hattori and coworkers studied the NIR-triggered TP release of nitroxide donors (2,2,6,6-tetramethylpiperidine-1-oxyl (TEMPO) radical) under air atmosphere (i.e., in the presence of O_2_) using the 2-(4-nitrophenyl)benzofuran (NPBF) caging group [73]. One- (365 nm) and two-photon (screening 710–760 nm wavelengths) release of the TEMPO radical was confirmed by electron paramagnetic resonance (EPR) spectroscopy (OP: 5 mM, 6.0 × 10^15^ photons/s, 2 probes: 80/56% TEMPO after 10 min/40 s irradiation (in benzene/DMSO) or 81/58% TEMPO after 20 min/40 s irradiation (in benzene/DMSO); TP: 10 mM in benzene, Ti:sapphire laser (pulse width 100 fs, 80 MHz, 700 mW), uncaging efficiency: 1.1 GM (740 nm)/0.22 GM (730 nm)). The cytotoxic activity of the released radicals was assessed in vitro in Lewis lung carcinoma (LLC) cells (trypan blue exclusion assay). With 360 nm light (1 min) vs. unirradiated controls, an irradiation-length- and cage-concentration-dependent cytotoxic effect was observed (100 μg/mL: 66.5% (after light irradiation) vs. 87.8% (without light irradiation) living cells). The role of photochemical radical generation in cytotoxicity was assessed by comparing the presence of reactive oxygen species (ROS) in irradiated and control samples (ROS-ID oxidative stress detection kit). ROS was detected in cells irradiated in cage-containing medium, however not in the unirradiated cage-containing samples or the samples irradiated without the cage. However, the low water solubility of the cages excluded further in vivo assays.

### 2.9. Thymidylate Synthase Inhibitors

Thymidylate synthase (TS) is a folate-dependent enzyme involved in the synthesis of 2′-deoxythymidine-5′-triphosphate. TS inhibitors are interesting as potential anticancer agents could be of a folate or nucleotide analog type. 5-Fluorouracil having a TS inhibitory effect is used in therapy since the 1960s for various GI, breast, and head and neck cancers. Lin et al. described a porphyrin-drug construct exploiting the tumor-affinity of porphyrins and using a PPG (an *o*-nitrobenzyl) for light-triggered drug release [74]. As anticancer model drug *tegafur* (a 5-fluorouracil prodrug) was selected, and as a negative control, a construct with uracil was prepared as well. Photolysis (350 nm) was studied with HPLC monitoring, showing 50% conversion in 12 min (Φ = 0.032). Cytotoxicity under dark and UV-irradiated conditions were studied on MCF-7 cells. In 50 µM concentration, *tegafur* led to 91% cell death. In the absence of light, the probe was modestly toxic (7% cell death); however, treatment with pre-irradiated samples led to 67% cell death, such as irradiation of cells treated with the probe (69% cell death). To ascertain that the cytotoxicity is due to photorelease and not singlet oxygen formation, a control irradiation experiment with uracil was run. The observed 6% cell death was comparable to the result obtained with UV light alone.

### 2.10. PROTACs (PROteolysis TArgeting Chimeras)

**Figure 11:**
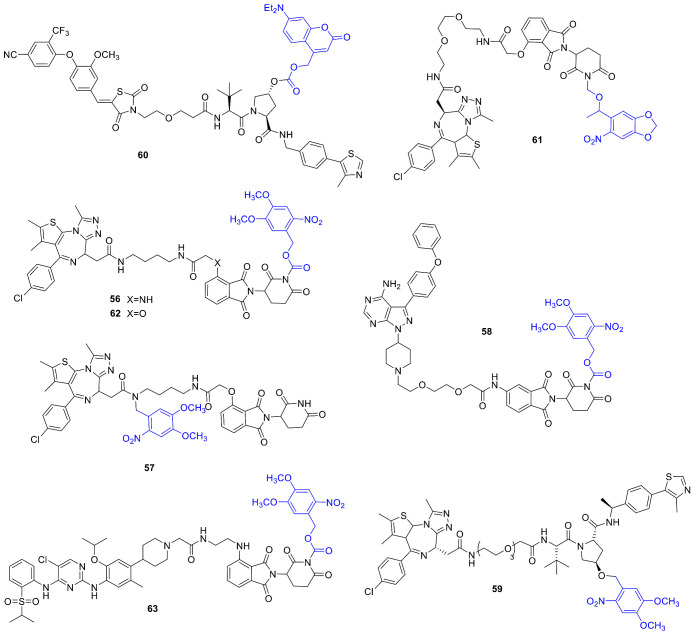
Photoactivatable PROTACs, the PPG indicated in blue.

Proteolysis targeting chimeras (PROTACs) emerged recently in the forefront of interest as a tool for inducing targeted protein degradation. PROTACs are bifunctional molecules that could facilitate binding between an E3 ligase and a protein of interest, thereby inducing protein degradation via the ubiquitin-proteasome pathway. With PROTACs formerly not druggable proteins could be addressed as well, including key players of oncogenic processes. The interest in PROTACs is also underlined by the fact that both photocage and photoswitch approaches have recently been disclosed [75,76]. Pan and coworkers developed photocaged PROTACs by linking a DMNB PPG to different positons of dBET1 (comprised of a *thalidomide* CRBN ligand and a JQ1 BRD4 ligand): either to the amide nitrogen of the JQ1 unit or the imide nitrogen of the *thalidomide* unit (**56**, **57**) (Figure 11) [77]. The target protein (BRD4) of dBET1 is a member of the BET (bromodomain and extraterminal) protein family and is acting via super-enhancers and oncogenes expression regulation. Several clinical studies with small-molecule BET inhibitors were initiated. Photolysis was studied with HPLC monitoring (365 nm, 3 mW/cm^2^), showing a rapid conversion of the probes (t_1/2_: **57** = 60 s, **56** = 105 s); however, dBET1 release only in the case of **57**. Binding assays confirmed efficient masking of activity upon PPG-linking (BRD4 IC_50_: **57** = 7.6 µM, JQ1 = 71 nM, dBET1 = 22 nM). BRD4 degradation was studied in Ramos cells under dark and UV-irradiated conditions (365 nm, 3 min). Upon light activation, **57** efficiently reduced BRD4 levels (D_max_ = 93% at 1 µM). Antiproliferative activity was studied on Namalwa cells in comparison with dBET1. UV irradiation could restore **57**’s activity to the same range as that of dBET1 (**57**-dark: GI_50_ = 3.1 µM, **57**-lit: GI_50_ = 0.4 µM, dBET1: GI_50_ = 0.34 µM). Long-term effects were studied in HUH7 cells (10-day colony-forming assay), confirming light-induced activation of **57** (almost complete inhibition at 5 µM vs. no effect on colony density under dark conditions). In vivo studies were conducted on the zebrafish embryo model (treatment at 12 h post fertilization, 10 min irradiation at 365 nm or dark conditions), with DMSO as blank and dBET1 as a positive control. Upon irradiation, **57** led to phenotypic changes similar to dBET1 as well as BRD4 degradation confirmed with Western blot analysis. The effect on BRD4 degradation could be visualized by injecting enhanced green fluorescent protein-tagged BRD4 into the embryos before treatments. The design strategy was extended to further targets, namely **58** was prepared in a similar fashion with linking DMNB to the imide nitrogen of MT-802, a BTK degrader. In Ramos cells, **58** led to BTK degradation in an irradiation-dependent manner.

Kounde et al. used an analog design for their photocaged PROTAC: a DMNB PPG attached to a critical OH of the E3 ligase ligand to prevent interaction and a JQ1 pan-bromodomain warhead connected with a PEG linker (**59**) [78]. Photocleavage was studied by UV/VIS and LC-MS monitoring at 365 nm (ACN/H_2_O 1/1, 50 μM, 25 mW LED), giving full conversion within 180 s. Cellular activity was assessed on HeLa cells, monitoring BRD4 levels with Western blot analysis. Incubation with the caged PROTAC followed by light irradiation (60 s, 25 mW 365 nm LED) led to dose-dependent protein degradation, not observed under dark conditions even after a 24 h incubation. Comparing the time frame of protein degradation between the parent drug and the caged following photorelease, similar results were obtained (i.e., the uncaging is not a rate-limiting step in the process). The overall phenotypic effect was tested with live-cell imaging prior to and after light irradiation on HeLa cells. The caged probe had a cytostatic effect (average proliferation fold change = 1); however, upon irradiation, similar reduced growth as with the parent agent could be obtained (average proliferation fold change < 0.5). The former effect is due to the in-cell target engagement (JQ1-BRD4) of the caged probe. The time course of light-activated protein degradation was measured by live-cell fluorescence imaging on HEK293 cells expressing GFP-BRD4, confirming a light-dependent effect for **59**.

Deiters and coworkers developed photocaged PROTACs for Von Hippel-Lindau (VHL) and CRBN E3 ligases [79]. For photoactivatable VHL ligands, the position of the PPG (a diethylamino coumarin (DEACM)) on the hydroxyproline was selected based on structural considerations (protein-ligand complex) (**60**). As a target, estrogen-related receptor α (ERRα) was chosen for the novel probe. ERRα is a member of the nuclear receptors superfamily, and its role in various (e.g., breast) cancers was confirmed by several studies. Photolysis assayed with HPLC and MS monitoring showed a clean release of the parent PROTAC. To challenge the efficiency of the caging, MCF-7 cells were treated with the probe under dark or irradiated conditions (365 nm, 3 min), and the ERRα levels were measured via Western blot analysis (after 8 h). Without irradiation, ERRα PROTAC **60** did not decrease ERRα levels; however, upon irradiation similar effect was recorded as for the parent PROTAC. The mechanistic pathway was confirmed with control experiments in the presence of the proteasome inhibitor MG132 or the neddylation inhibitor MLN4924. For designing a photocaged CRBN ligand, the PPG (6-nitropiperonyloxymethyl, NPOM) was linked to the imide moiety of the glutarimide ring (**61**) of the parent molecule. In addition, in this case, photorelease was confirmed by HPLC and MS analysis. BRD4 degradation was studied in HEK293T cells with Western blot analysis. Photocaging efficiently masked the activity of the parent PROTAC, which could be unleashed with UV irradiation (365 nm, 180 s). The proteasome- and E3-ligase mediated pathway was confirmed with control experiments with MG132 and MLN4924. To assess the effect of BRD4 degradation, 22Rv1 cells (castration-resistant prostate cancer cell line) were treated with the parent drug (72 h: 51% reduced cell viability) and **61** with and without light activation (**61**-dark: no significant effect, **61**-lit: 72 h, 39% reduced cell viability). Monitoring caspase-3/7 activation, a similar effect was observed for the parent drug and light-activated **61**.

Liu et al. started their investigations on a parent lead compound: *pomalidomide,* linked to a Nvoc PPG to block its binding to E3 ligase CRBN (coined opto-*pomalidomide* by the authors) [80]. The attachment point of the PPG at the glutarimide NH was selected based on structural considerations. Photorelease at 365 nm was confirmed with UPLC-MS and UV/VIS monitoring (t_1/2_ = 62 min). Opto-*pomalidomide* lost in vitro CRBN binding that could be regained upon 365 nm irradiation (competitive binding assay). Light activation of opto-*pomalidomide* intracellularly (on HEK293T cells) was studied via its effect on the protein-protein interaction between CRBN and IKZF1 (Ikaros zinc finger transcription factor) and CRBN-dependent IKZF1/3 degradation. The activity of opto-*pomalidomide* could be unleashed upon UV irradiation (15 min, no effect without). Exposing opto-*pomalidomide* pretreated cells to different lengths of UV irradiation, a light- and drug dose-dependent IKZF1/3 degradation was recorded (not induced by UV irradiation itself). The effect of opto-*pomalidomide* on multiple myelome cells was also light- and CRBN-dependent (dark conditions vs. 15 min 365 nm irradiation, MM.1S cell proliferation, CCK-8 cell viability assay). Opto-*pomalidomide* was used for the synthesis of two light-activatable PROTACs: opto-dBET1 (**62**) and opto-dALK (**63**). Opto-dBET1 lost the parent drug activity (ubiquitination of BRD2/3) due to caging; however, the effect could be restored intracellularly with UV light (15 min 365 nm irradiation, HEK293T cells). Subjecting **62** pretreated HEK293FT cells to 5 or 15 min UV irradiation, a CRBN and ubiquitin-proteasome system-dependent BRD3/4 degradation could be operated. While **62** itself is inert, light activation could offer precision control for avoiding future toxicity issues stemming from complete protein depletion. A dose-dependent effect of **62** on cell proliferation was recorded (HEK293FT and C4-2 cells, CCK-8 cell viability assay). The second target, ALK (anaplastic lymphoma kinase), was selected due to the presence of ALK fusion proteins in several cancer types (e.g., non-Hodgkin’s lymphoma). As for opto-dBET1, first, the in vitro UV photorelease of opto-dALK was confirmed (UV/VIS monitoring). Opto-dALK was inactive in ALK degradation; however, with UV irradiation, a light- and drug dose-dependent EML-ALK degradation was observed (NCI-H2228 or NCI-3122 non-small cell lung cancer cell lines). Light activation of **63** also promoted NPM-ALK fusion protein degradation in an anaplastic large cell lymphoma cell line (SU-DHL-1). While UV irradiation itself did not have a considerable effect, following uncaging **63** reduced the viability of NCI-H2228, NCI-3122, and SU-DHL-1 cells. Given the sensitivity of SU-DHL-1 cell growth to NPM-ALK degradation, the cell proliferation effect was more pronounced on this model system.

### 2.11. Modulators of p53 Signaling

p53 is a central tumor suppressor. Its intracellular level is thoroughly regulated. p53 mutations were identified in several cancers. MDM2 (mouse double minute 2 homolog) protein acts as a negative regulator of the p53 pathway (via a protein-protein interaction) and was found to be overexpressed in different cancer cell lines. For selective reactivation of p53 signaling, Hansen et al. designed a photoactivatable MDM2 inhibitor starting from idasanutlin and using a coumarine PPG [81]. Based on structural considerations (SAR and docking studies), the PPG was linked to the *m*-methoxybenzoic moiety of the pharmacological agent. Photolysis (400 nm, physiological conditions, 0.1% quantum yield) cleanly afforded the free idasanutlin, as monitored by UV/VIS and UPLC-MS, and the construct showed suitable hydrolytic stability. The light-triggered activity of PPG-idasanutlin was studied on p53-proficient retinal pigment epithelial cells (RPE-1) in comparison with DMSO control, nutlin-3, and the free idasanutlin with or without 400 nm light (immunofluorescent staining of nuclear p53 protein levels). PPG-idasanutlin showed activity only upon light activation (increased p53 level); moreover, a dose-dependent effect could be attained by varying the duration of the 400 nm light irradiation or the PPG-idasanutlin dose. The novel probe efficiently blocked the colony outgrowth of RPE-1 cells following exposure to 400 nm light (no similar effect with DMSO+400 nm light control experiment); however, it had no effect without light irradiation. The scope of the approach was ascertained on further nontransformed (BJ-hTert) and tumor (RKO, U2OS) cell lines. Selective activation of PPG-idasanutlin with the single-cell resolution was demonstrated in a 2D cell culture setup of RPE-1 cells expressing venus-tagged p53. Short (0.1 s) 405 nm laser pulses of selected regions (5 μm interspaced positions) led to a pharmacological effect confined to the irradiated areas, as observed by monitoring the cell cycle progression and quantifying the nuclear p53-venus signal. Diminished effect in neighboring cells (due to diffusion of the photoreleased active agent) and no effect in unirradiated cells in adjacent wells were recorded.

## 3. Photoswitches for Antitumor Applications

As the concept of photocages, that of photoswitches also dates back to several decades [82], however particularly the recent years have seen a surge of activity in the field (referred to as photopharmacology) [83,84]. The application of photoswitchable pharmacological agents is based on their two (or more) interconvertible isomeric forms that allow significant steric changes upon photoisomerization and, consequently, different pharmacological activity. The photoisomerization could proceed via two principal routes, *trans*→*cis* (*E*→*Z*) isomerization (e.g., azobenzenes and their heteroaromatic analogs, indigos, hemithioindigos, stilbenes, hydrazones, and iminothioindoxyls) or 6π electrocyclization of a triene system (diarylethenes) [85]. Of further types, spiropyrans, fulgides, and donor-acceptor Stenhouse adducts were exploited [86]. Importantly, the photoisomerization process is reversible, that is the basic difference vs. photocages. Although the reversibility confers many advantages, it also adds further layers of complexity. Selective irradiation is feasible in the case of appropriate band separation, and for further biological applications, the activation wavelengths should ideally be in the biological window. For therapeutic applications, the probes should have ideally high photofatigue resistance, rapid isomerization kinetics, a photostationary state (PSS) sufficiently enriched in the active isomer, as well as a half-life of the metastable state in line with the planned application. Depending on the target and the probe’s structure, different scenarios are possible. Both the thermodynamically more stable (dark) or the less stable (obtained upon light activation) form could have higher bioactivity. Typically, the sought-for scenario is where the active form is obtained upon irradiation (i.e., a turn-on activity); thereby, administration problems could be avoided or background activity resulting from incomplete photoisomerization. The active form could isomerize back to the inactive isomer thermally (T-type photoswitches, thermally reversible) or upon light irradiation (P-type photoswitches, photochemically reversible), therefore a more localized effect could be obtained, unlike as for the photocages, where diffusion of the active form from the site of effect might raise concerns. Besides the optimal photophysical and photochemical properties, the novel probes should also comply with the criteria of pharmacological/therapeutic applications, as lack of ground state toxicity, feasible hydrolytic solubility and stability, and resistance toward reduction in biological media (e.g., by glutathione) [87]. The general strategy for designing a reversibly activatable drug molecule is either to add a photoswitchable (often arylazo→“azo-extension”) tag to the pharmacophore of the parent structure or to incorporate a (often arylazo→“azologization”) photoswitchable unit into the pharmacophore. The latter strategy could be expected to alter less the overall structure, therefore having less impact on the pharmacokinetic and pharmacodynamic properties of the parent drug. Evidently, for both approaches besides a clinically validated target, detailed SAR and structural information are necessary of the target and the parent drug (family). Finally, from a practical point of view, synthetic accessibility of the novel probes, availability of straightforward synthetic methods for the desired modifications should also be duly considered.

### 3.1. Photoswitchable Kinase Inhibitors

**Figure 12:**
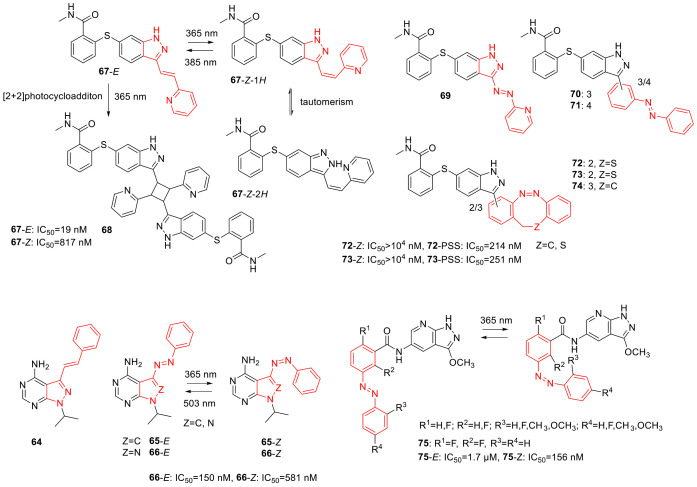
Photoswitchable kinase inhibitors, the photoswitch unit indicated in red.

One of the first photoswitchable kinase inhibitors, a REarranged during Transfection (RET) tyrosine kinase inhibitor, was described by Grøtli and coworkers in 2015, based on an azobenzene-derived pyrazolopyrimidine scaffold and its stilbene counterpart (**64**–**66**) (Figure 12) [88]. The photoswitching properties of the novel probes were studied with UV/VIS monitoring. The stilbene derivative **64** underwent an irreversible photoreaction at 302 nm; however, the azobenzene derivatives (**65**, **66**) photoisomerized reversibly (**65**: 365 nm *E*→*Z*, thermal half-life: τ = 2.0 min (37 °C); **66**: 365 nm *E*→*Z*, PSS: 87% *Z*, thermal half-life: τ = 9.7 h (37 °C), 503 nm *Z*→*E*). The probes showed appropriate hydrolytic stability and resistance to photofatigue upon 10 photoswitching cycles. Assessing in vitro RET kinase activity under dark and lit conditions (365 nm, 3 min), a dose- and illumination-dependent effect was recorded (**66**-*E*: IC_50_ = 150 nM, **66**-*Z*: IC_50_ = 581 nM). The in vitro results were corroborated by cellular activity assays (**66**-*E*: IC_50_ = 3.8 µM, **66**-*Z*: IC_50_ = 12 µM) after ascertaining the irradiation tolerance of the study system (365 nm, 15 min). However, despite the important proof-of-concept value of the study, for therapeutic applications, the decrease in activity upon irradiation is generally a less sought-for scenario.

Peifer and coworkers studied the photoisomerization of the approved (for renal cell carcinoma) ATP-competitive, type II tyrosine kinase inhibitor *axitinib*, targeting VEGFR2 (**67**) [89]. *Axitinib* has a stilbene-like moiety in its structure; moreover, instances for its photoinduced isomerization were reported. As a starting point to their studies, Peifer and coworkers examined by molecular modeling the binding modes of the *E*- and *Z* isomers of *axitinib* in the ATP pocket of VEGFR2. The *Z* isomer leading to implausible binding modes upon docking was assumed to be an inactive form. For further studies, *Z*-*axitinib* (**67**-*Z*) was prepared from *E*-*axitinib* (**67**-*E*) by 365 nm irradiation; however, the structural analysis revealed the presence of two tautomeric (**67**-*Z*-1*H* and **67**-*Z*-2*H*) forms. Photoisomerization and photostationary states were studied in DMSO and water at different wavelengths by HPLC-analysis and ^1^H NMR (PSS (365 nm) (%) in DMSO: **67**-*E* 40/**67**-*Z* 60; PSS (385 nm) (%) in DMSO: **67**-*E* 78/**67**-*Z* 22; PSS (385 nm) (%) in water: **67**-*E* 96/**67**-*Z* 4), showing a reversible but incomplete process in DMSO and an irreversible, but complete *Z*→*E* process in water. *Axitinib* did not show significant photofatigue in DMSO following several switching cycles; however, at 365 nm in the water, a competing dimerization occurred (formation of **68**, not observed at 385 nm throughout the *Z*→*E* conversion). The two isomers were subjected to in vitro VEGFR2 kinase assay and selectivity profiling in a panel of 300 kinases, revealing surprisingly similar IC_50_ values and selectivity (VEGFR2—**67**-*E*: IC_50_ = 25 nM, **67**-*Z*: IC_50_ = 65 nM). Re-running the assays (VEGFR2 and PDGFRβ) under controlled light conditions showed a 43-fold difference between the isomers, as predicted by the *Z* isomer being less active. IC_50_ values were determined in VEGFR2 kinase assay for both isomers following UV irradiation (*Z*: 385 nm irradiation, *E*: 365 nm irradiation). The IC_50_ observed upon irradiating the *Z* isomer (IC_50_ = 29 nM) suggested an almost complete *Z*→*E* conversion in vitro; however, in the case of the *E* isomer, a more complex reaction occurred (IC_50_ = 44 nM), leading to a mixture containing both isomers and the photodimer product **68** (not active itself in VEGFR2 kinase assay). For cellular studies, a HUVEC (human umbilical vein cells) proliferation assay was selected as a model system for angiogenesis. Dose-response curves were measured in the dark, showing a 31-fold difference between the isomers (**67**-*E*: IC_50_ = 0.83 µM, **67**-*Z*: IC_50_ = 26 µM). Irradiating the Z isomer treated cells at 385 nm (after ascertaining the UV-tolerance of the system, 5 min irradiation) led to a similar inhibition curve to that of the *E* isomer, therefore suggested an efficient photoswitching. The difference in activities was further confirmed by Western blot analysis in HUVECS and NIH/3T3 cells, studying VEGFR2 and AKT phosphorylation. Kinase selectivity profiling of the two isomers was re-run under controlled light conditions using HUVECs (by PamGene PamstationR and PamChipR). Besides identifying three eligible target tyrosine kinases for **67**-*E* (FGFR1, RET, SYK), **67**-*Z* was found to act on the relevant kinases with a decreased intensity. Importantly, the study method used could be an alternative of high-throughput commercial kinase selectivity screening typically carried out at ambient light conditions, which might be a serious concern for photoactivatable compounds.

In a follow-up report, the same group set out to prepare azologized versions of *axitinib*, incorporating an azobenzene (**69**–**71**) or a diazocine unit (**72**–**74**) into the parent structure to solve isomerization reversibility issues [90]. Based on previous results with *axitinib*, the *E*-azoaxitinibe **69** was assumed to be the active form (attaching the azo group directly to the indazole ring). However, as no photoswitching was observed for the azologized probe (UV/VIS monitoring) due to fast thermal back-isomerization (due to azo-hydrazone tautomerization) and also the bioactivity was diminished vs. the parent drug, this design was dismissed. As a second approach, bistable (sulfur)-diazocine derivatives were designed (**72**–**74**), presumably activated upon light irradiation in their *E* form (backed up by docking analyses) and compared with two azobenzene derivatives (**70**, **71**). Photochemical characterization was run in DMSO solution with UV/VIS and ^1^H NMR monitoring (PSS (*E*/*Z*%)—**70**: 17/83 (365 nm), **71**: 20/80 (385 nm), **72**: 47/53 (405 nm), **73**: 25/75 (405 nm), **74**: 60/40 (405 nm); t_1/2_ (h), 37 °C—**70**: 43, **71**: 5.7, **72**: 7.3, **73**: 3.7, **74**: 1.5). For diazocines, a lower photoconversion was observed; however, with 530 nm irradiation, a quantitative back-isomerization could be operated. No significant photofatigue was detected till 20 cycles (alternating 20 s irradiation at 405/530 nm), and the half-lives were sufficiently long for in vitro assays. Bioactivity was assessed first in VEGFR-2 kinase assay under controlled light conditions (IC_50_ (nM)—**69**-*E*: 415, **70**-*E*: 1077, **70**-PSS: 1289, **71**-*E*: 1020, **71**-PSS: 1435, **72**-*Z* > 10,000, **72**-PSS: 214, **73**-*Z* > 10,000, **73**-PSS: 251, **74**-*Z*: n/a, **74**-PSS: 493). As expected from the modeling results, the azobenzene derivatives were moderately *E* active without significant difference between the isomeric forms. For the diazocine derivatives, irradiation increased significantly the activity (40–47-fold), even despite the relatively low *E*-content of the PSS. Compound **72** was subjected to kinase profiling (PamGene, HUVECs lysates). Prior irradiation **72** remained inactive, whereas, after irradiation, a moderate effect was observed. Of note, the water solubility of the novel probes should be further optimized (necessitating a DMSO-cosolvent for assays).

Szymanski and coworkers developed reversibly photoactivatable BRAF^V600E^ kinase inhibitors, using an analog of the clinically approved BRAF^V600E^-selective ATP-competitive inhibitor *vemurafenib* as the starting point and an azobenzene photoresponsive unit [91,92]. The parent inhibitor had an amide and a sulfonamide linker, which both might be suitable for azobenzene replacement. However, studying co-crystal structures of inhibitors binding BRAF^V600E^, it was found that the arrangement of the aromatic rings linked by the amide moiety would resemble more to an *E* azobenzene isomer endowing it presumably with higher activity. On the other hand, a bent conformation around the sulfonamide pointed toward the *Z* azobenzene isomers being more active. As *Z* isomers being more potent is the sought-for scenario for therapeutic uses (allowing an on-demand turn-on activity), the photoswitch unit was introduced at the sulfonamide. Eight novel derivatives were prepared with the aim of studying the effect of different substitution patterns on bioactivity and the main photochemical parameters (main absorption band, rate of thermal relaxation, *Z*/*E* ratio in the PSS). PSS composition was monitored by ^1^H NMR spectroscopy, appropriately placed methoxy substituents leading to nearly quantitative photoisomerization (≥88% *Z* at PSS). *Z* isomer half-lives were monitored in DMSO and BRAF assay buffer/or BRAF assay buffer-ACN mixture, giving values in the hours range (DMSO: >10–70 h, BRAF assay buffer/or BRAF assay buffer-ACN: 5 to >24 h). The bioactivity for both isomers was screened on purified recombinant BRAF^V600E^, quantifying the phosphorylated-MEK-1 product with a Western blot to assess the potency vs. the parent compounds and the difference in activity between the dark and lit states, as well as allowing SAR considerations. The most promising derivative (**75**) showed a 10-fold difference of activity between the two isomers (*vemurafenib*—IC_50_ = 9.6 nM, parent inhibitor—IC_50_ = 22 nM, **75** (dark, *E*)—IC_50_ = 1.7 µM vs. **75** (lit, *Z*)—156 nM), with a reversible switch of activity upon illumination and relaxation. These results were in accordance with binding modes obtained upon docking of the inhibitor into the crystal structure of BRAF^V600E^. Cellular cytotoxicity assays were performed on A375 cells (cell viability MTS assay), typically used for BRAF^V600E^ inhibitors; however, the enzymatic assay activity could not be translated onto this system (i.e., no activity was observed in neither states). As azologization of a selective kinase inhibitor might result in a loss of selectivity, the kinome inhibition profile of **75** was recorded to check for eventual off-target activities potentially explaining the cellular inactivity (on cell lysates from SK-Mel-28 cells). Increased phosphorylation levels were found compared to the DMSO control; moreover, **75** has a less favorable PSS ratio (55% *Z*) that could also be an important factor of modest cellular performance.

**Figure 13:**
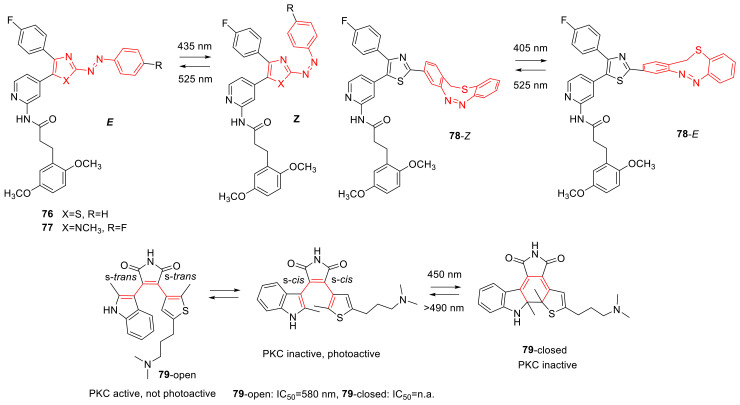
Photoswitchable kinase inhibitors, the photoswitch unit indicated in red.

Herges and coworkers reported novel photoswitchable p38α Mitogen-activated protein kinase (p38α MAPK) and Casein Kinase 1δ (CK1δ) inhibitors [93] and revealed potential artifacts resulting from pharmacological assay conditions. Using a known 4,5-diarylimidazole inhibitor as the starting point [94], the potential modification site was designed via docking studies. The 5-membered aromatic ring of the original inhibitor was designed into the photoswitchable unit; however, to optimize the photoisomerization process was modified either into a thiazole or an *N*-methylimidazole ring (Figure 13). Besides an azobenzene (**76**, **77**), a diazocine photoswitchable unit was tested as well (**78**), in the latter case (unlike as for azobenzenes), the *Z* isomer being thermodynamically more stable. The docking studies confirmed significant differences in the potential binding of the *E*- and *Z* isomers for both structural types; therefore, a difference in their pharmacological activities was expected as well. Throughout the photochemical characterization of the products (in DMSO due to solubility reasons, UV/VIS absorption, photostability, PSS ratio, and half-lives, alternating isomerization), **76** showed concentration-dependent photoisomerization (~80% *E*→*Z* vs. 29% at 3.5 mM and no conversion at 17 mM, no concentration-dependence in the 5 µM-100 µM range), that was ascribed to aggregation effects, as well as an unfavorably short half-life. Compounds **77** and **78** showed no such concentration dependency (**76**: PSS—29% *Z* (435 nm), 81% *E* (525 nm), t_1/2_ = 13 min; **77**: 85% *Z* (420 nm), 88% *E* (525 nm), t_1/2_ = 2.4 h; **78**: 47% *E* (405 nm), 100% *Z* (525 nm), t_1/2_ = 3.2 d). The pharmacological characterization of the diarylazo products did not confirm the expected tendencies, while the diazocine showed no activity (in vitro kinase assay: CK1δ—**76**: *E* (dark) IC_50_ = 147 nM, PSS (435 nm) IC_50_ = 55 nM; **77**: *E* (dark) IC_50_ = 138 nM, PSS (420 nm) IC_50_ = 218 nM; **78**: not active up to 10 µM; p38α: **76**: *E* (dark) IC_50_ = 29 nM, PSS (435 nm) IC_50_ = 2.4 nM; **77**: *E* (dark) IC_50_ = 83 nM, PSS (420 nm) IC_50_ = 115 nM; **78**: not active up to 10 µM). To explain the observed discrepancies, further studies (co-crystallization of **76** in complex with p38 MAPK under ambient conditions, dithiothreitol (DTT) treatment with NMR monitoring) revealed the formation of a reduced hydrazine product from **76** due to the DTT present in the assay system. To rule out the effect of the reduction, p38α kinase assay was re-run without DTT (**76**-*E* (dark): IC_50_ = 67 nM, **76**-PSS (435 nm): IC_50_ = 105 nM), however CK1δ without GSH could not be performed. Co-crystallization of **76** and **77** with CK1δ without DTT was performed, confirming the presence of the diazo moiety; however, no stable conformation was adopted that might explain the modest differences of activity upon isomerization. As the susceptibility to reduction is structure-dependent (no reduction observed for **77**), it should be duly considered in the design of novel photoswitchable agents. On the other hand, reducing and stabilizing agents often used in biological assays might lead to artifacts, such as ambient light conditions. Further points of consideration are the aqueous solubility of the compounds and their photophysical properties in aqueous media (PSS, *E*/*Z* half-lives).

Branda and coworkers studied the design of photoactivatable protein kinase C (PKC) inhibitors starting from the bisindolylmaleimide family of ATP-competitive protein kinase inhibitors and using a diarylethene photoswitch scaffold [95]. Protein kinase C isoforms are involved in pathways related to the expression of genes affecting cell cycle progression, tumorigenesis, and metastatic dissemination. In the studied design, the maleimide ring of the parent structures with the indole heterocycles at C3 and C4 contains the 1,3,5-hexatriene motif required for the photochemical ring-closing; however, it is not adopting a photochemically productive conformation. To obtain a photoswitchable analog, one indole was replaced with a thiophene (**79**). Consequently, the PKC-active form could rotate into the *s-cis-s-cis* conformer and undergo cyclization into the PKC-inactive form (open→closed: 450 nm, PSS: 42% closed form after 7 min irradiation at 50 µM, 0.7 mW/cm^2^; closed→open: >490 nm). The photoinduced ring-closing reaction was highly solvent-dependent and not operational in polar solvents (e.g., DMSO, water), the reverse ring-opening reaction not suffering from these limitations. In vitro activity was studied on PKC βII (Z’-Lyte assay kit), confirming lack of activity for the ring-closed isomer vs. a potent activity of the open form (open: IC_50_ = 580 nM, closed: no measurable dose-response trend). The activity in the assay could be restored upon light irradiation (buffered solution of PKC and the closed form in 0.2–25 µM concentration, 30 s irradiation at >490 nm).

### 3.2. Photoswitchable Epigenetic Modulators

**Figure 14:**
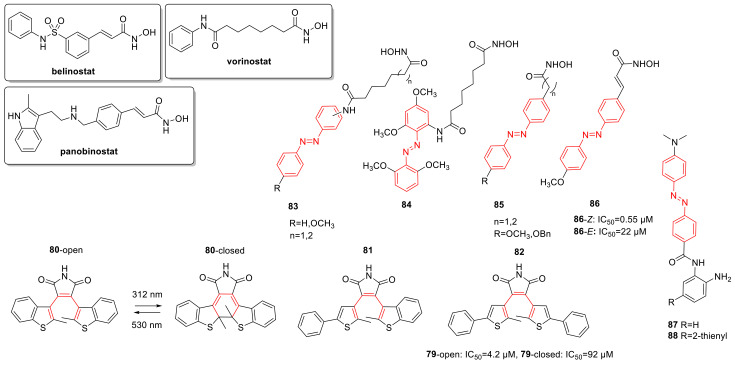
Photoswitchable HDAC inhibitors, the photoswitch unit indicated in red.

König and coworkers combined a diarylethene photoswitch and a bisindoylmaleimide pharmacophore to obtain photoactivatable sirtuin inhibitors (3 novel probes: symmetrical benzothiophene **80** and replacement of one (**81**) or both benzothiophenes by phenyl substituted thiophenes (**82**)) (Figure 14) [96]. Photoswitching at 312 nm was studied in DMSO (10 µM) with UV and HPLC monitoring (PSS, %: **80**—62, **81**—87, **82**—94), showing a clean process and resistance to photofatigue (over 5 cycles). In vitro activity against sirtuins was evaluated in a fluorescent assay (ZMAL) (IC_50_ (µM), hSirt2—**82**-open = 4.2, **82**-closed = 92; **81**-open = 2.3, **81**-closed = 2.1; **80**-open = 13; **80**-closed = 23). Two probes (**81**, **82**) showed activity in the range of the reference inhibitor Ro31-8220 (IC_50_ = 0.80 µM); however, for **81,** no difference between the open and closed forms was registered. Compound **82,** however, besides a 20-fold difference in activity between the open and closed forms, showed also isotype selectivity toward hSirt1 and hSirt3. The in vitro results were cross-examined with molecular dockings on human sirtuins. To confirm in situ activation, the closed form was irradiated under the assay conditions (30 min, 2.5 W, 530 nm), and the time course of the inhibition was monitored. A constant inhibition was obtained over 20 min, with a value corresponding to that obtained previously with the open form. The cellular activity of **82** was tested via tubulin hyperacetylation, showing similar tendencies as the Ro31-8220 reference compound.

Feringa and coworkers studied the design of photoswitchable HDAC inhibitors [97] using as starting point *vorinostat* (SAHA), *panobinostat,* and *belinostat* (inhibitors approved for clinical use), and azobenzene as the photoswitch unit. Using structural knowledge on the parent drug (*vorinostat*), two molecular designs were envisaged, introducing the photoswitch either to the cap (**83**, **84**) or the linker region (**85**, **86**). To study their influence on the photochemical and pharmacological properties, different attachment positions and substitution patterns on the azobenzene were assessed in the former, whereas different linker lengths and types in the latter approach. All novel compounds showed appropriate PSS values (above 75%) upon 365 nm irradiation in DMSO (determined by ^1^H NMR spectroscopy). Depending on the substitution pattern, the half-life of the *Z* isomer spanned from the minutes to the hours range (determined by UV/VIS spectroscopy at rt, in HDAC assay buffer with 1% DMSO), an important property for auto-inactivation upon therapeutical use. The first screen of activity was performed on class I HDACs (HeLa nuclear extracts), human recombinant class I HDACs (1–3, 8) and HDAC6. Based on the pharmacological results, compound **86** was selected for further studies, due to a potent activity of the *Z* state (HDAC1 (*E*): IC_50_ = 0.66 µM, HDAC1 (*Z*): IC_50_ = 0.080 µM; HDAC2 (*E*): IC_50_ = 22 µM, HDAC2 (*Z*): IC_50_ = 0.55 µM, HDAC3 (*E*): IC_50_ = 0.32 µM, HDAC3 (*Z*): IC_50_ = 0.071 µM; *vorinostat*: HDAC2—IC_50_ = 0.18 µM), appropriate *Z*/*E* activity ratio and reversible action upon illumination. Cytotoxicity of selected compounds was measured in a cellular assay (cell viability of HeLa cells) in both isomeric forms. For **86**, in line with the HDAC assays, the *Z* isomer showed higher toxicity.

Reis et al. addressed the concept of using photoswitchable small-molecule ligands with fast equilibrium kinetics and long target residence times in the context of HDAC inhibitors [98]. For the probe design, the DABCYL fluorescence quencher and known *ortho*-anilide HDAC inhibitors (CI-994, Merck C60, MS-275) with a class I HDAC (1–3) preference were used as starting points (2 novel probes (**87**, **88**), a control probe and an HDAC1/2-biased probe were prepared). The expected isomerization-dependent activity was rationalized with computational studies, as well as structural and SAR information. Differential zinc-chelating ability was expected from different electronic properties rather than resulting from the steric change. Thermal relaxation half-lives were measured under physiological conditions with microsecond resolution (55–60 μs), however were expected to still allow subcellular activation vs. diffusion. Light intensity- (470 nm, I_max_ = 17 mW/cm^2^) and concentration-dependent HDAC inhibitory activity of the novel probes against HDAC1/2/3 were measured. The observed selectivity and light-dependence of activity were in accordance with the previous design and mechanistic considerations (the authors coined the overall approach “chemo-optical modulation of epigenetically regulated transcription” (COMET)). Target residence times and dark phase recovery of enzymatic activity were addressed with different irradiation-dark period regimes. Cellular activity of the novel probes was measured in MCF-7 cells via acetylation of histone H3 lysine (H3K9ac) as a marker, confirming a light-dependent action. Histone H3K9ac levels remained elevated even after 8 h under dark conditions, in line with the long residence time and prolonged HDAC inhibition of the probes. Spatial control of activity (<1 mm) was confirmed by differential light exposure of treated MCF-7 cells (differential histone H3K9ac depending on light exposure). Slight histone H3K9ac increase in non-exposed areas was ascribed to suboptimal light-delivery processes. The effect on the transcriptome was assayed under various experimental conditions (dose, treatment duration, light exposure) with the L1000 assay method on MCF-7 cells. Expression patterns observed with **87** under light exposure and CI-994 supported a similar activity. To gain a further mechanistic insight, network analysis of on- and off-target genes was performed.

### 3.3. Photoswitchable Antimetabolites

**Figure 15:**
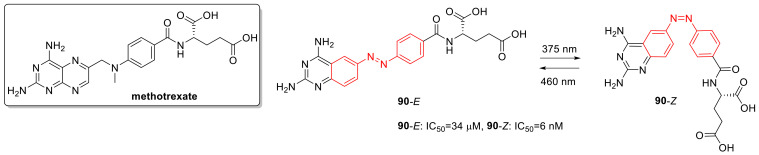
Photoswitchable *methotrexate* analog, the photoswitch unit indicated in red.

Gorostiza and coworkers studied photoswitching of the human dihydrofolate reductase (DHFR) inhibitor *methotrexate* (MTX), selected following stringent chemical-clinical-(photo)pharmacological criteria [99]. DHFR catalyzes a key step of folate metabolism, the conversion of dihydrofolate to tetrahydrofolate. Disrupting folate synthesis impairs thymidine and purine synthesis, consequently the synthesis of DNA, RNA, or proteins, therefore could reduce cell growth and proliferation. MTX could be used alone or in combination against, e.g., breast cancer, bladder, or lung cancer. However, MTX is also characterized by serious adverse effects (e.g., ulcerative stomatitis, leukopenia), necessitating dose reduction or treatment discontinuance. Based on the target binding of *methotrexate*, a higher activity for the thermodynamically less stable *Z* isomer could be expected (docking studies); moreover, the drug structure is feasible for the azologization approach (Figure 15). The appropriate site for introducing an azo group was selected based on synthetic and SAR considerations, i.e., executing tolerated modifications/isosteric replacements. The presumed activity difference of the isomers was backed up by molecular docking (human DHFR). The novel analog, called phototrexate by the authors (**90**), was obtained by isosteric replacements of the N5 and N8 nitrogens of the pteridine ring and introducing a diazo bond at C6 of the resulting quinazoline. *E*→*Z* photoisomerization could be efficiently effectuated at 375 nm, *Z*→*E* isomerization at 460 nm, without significant photofatigue. The ratio of the isomers at the photostationary phase was determined by ^1^H NMR (76% *Z* isomer after 375 nm irradiation, 30 min, 4.6 J/cm^2^, maximal photoconversion could be achieved with a fluence > 0.6 J/cm^2^). Thermal *Z*→*E* isomerization half-life under dark conditions was found to be 236 min, phototrexate is a T-type photoswitch (37 °C, aq. solution). Regarding the pharmacological activity, the *Z* isomer showed a stronger inhibition effect on DHFR (colorimetric assay), in accordance with the docking results (*Z*: 70% inhibition at 10 nM, *E*: 21% inhibition at 10 nM). In a cellular assay of cytotoxicity (MTT assay of cell viability, HeLa cells), the same tendencies were observed (*Z*: IC_50_ = 6 nM, E: IC_50_ = 34 μM). Of note, the difference in pharmacological activity and efficient in situ photoisomerization was further studied in vivo using zebrafish larvae (monitoring viability, anatomical and behavioral traits, comparison at 72 and 96 h post fertilization) using *methotrexate* as a positive control. Light-illumination (twice a day with 375 nm light, 0.61 J/cm^2^, 4 min irradiation) led to a 2- and 8-fold increase in abnormality and mortality for the *Z* vs. *E* isomer (abnormalities at 96 h: *E*—30%, *Z*—60%; mortality: *E*—6% of the total, *Z*—46% of total). Minor discrepancies between the activity of the parent drug and the active *Z* isomer on the pharmacological assays were ascribed to the different off-target activity profiles.

### 3.4. Photoswitchable PAD Inhibitors

**Figure 16:**
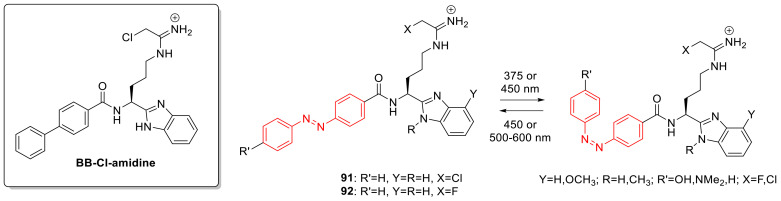
Photoswitchable PAD inhibitors, the photoswitch unit indicated in red.

Protein arginine deiminase (PAD) is involved in the conversion of arginine to citrulline. PAD4 was found to be overexpressed in several cancer cell lines (e.g., breast carcinoma, hepatocellular carcinomas); therefore, a role for altered PAD4 activity in the pathogenesis was suggested. Mondal et al. studied photoactivatable PAD inhibitors, using the known inhibitor BB-Cl-amidine as their starting point [100]. As BB-Cl-amidine contains a biphenyl moiety in its structure, it was assumed that replacing it with a photoswitchable azobenzene unit might be a feasible approach. Eight novel derivatives were prepared, modifying the amidine warhead and the substitution pattern of the azobenzene to modulate the enzyme binding affinity and the PAD isoform-specificity (Figure 16). Photoisomerization was studied by UV/VIS spectroscopy, ascertaining the thermal stability of the thermodynamically less stable Z isomer (compound **91**-*E*: > 12 h half-life) and a fast *E*→*Z* conversion upon irradiation (320–380 nm, **92**-*E*: > 80% conversion after 40–60 s). The *E* isomer could be photoconverted back (400–450 nm); however, this process was of less interest as the studied compounds act as irreversible inhibitors on the target. The bioactivity of the isomers was assessed in vitro in PAD-inhibition (PAD1-4) assays (COLDER assay). Two promising compounds were identified for photocontrolling PAD-2 activity (implicated in breast cancer). *E*→*Z* isomerization of **91** led to a 10-fold increase in activity, whereas *E*→*Z* isomerization of **92** led to a 45-fold deactivation. For **91,** similar results were obtained in a competitive activity-based protein profiling test (using PAD-targeted rhodamine-conjugated fluoroamidine labeling), however similar IC_50_ values were recorded for the two forms of **92** despite the previously observed activity difference (**91:** *E* IC_50_ > 100 µM, *Z* IC_50_ = 9.1 µM; **92**: *E* IC_50_ = 12 µM, *Z* IC_50_ = 24 µM). To elucidate this result, further enzyme kinetics studies suggested **92** to act as a reversible, competitive inhibitor (K_i_ = 25 μM). Cellular activity was assessed by studying histone H3 citrullination in HEK293T/PAD2 overexpressing cells. Upon photoactivation, **91**-*Z* showed a dose-dependent citrullination inhibition activity (the *E* isomer showing no activity at 100 µM), whereas **92**-*E* was only marginally active (~33% inhibition at 100 µM) and its *Z*-counterpart inactive. Regarding its cytotoxicity on HEK293T/PAD2 cells, **91** had a similar effect as the parent BB-Cl-amidine, however with no difference between the *Z* and *E* isomers.

### 3.5. Microtubule-Targeting Agents

**Figure 17:**
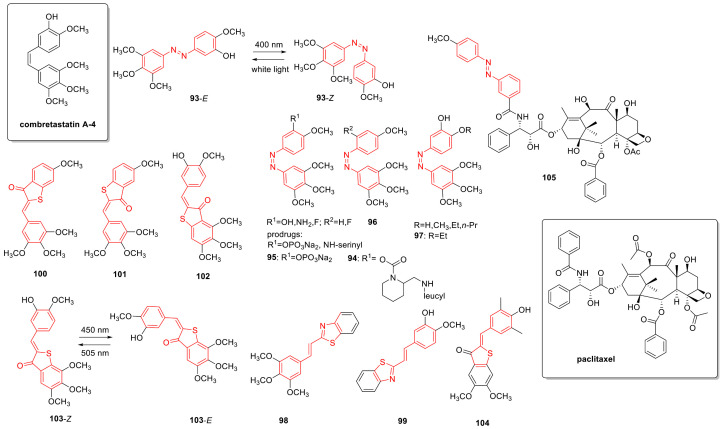
Photoswitchable microtubule-targeting agents, the photoswitch unit indicated in red.

Small-molecule drugs affecting MT dynamics could exert antimitotic and pro-apoptotic effects. Combretastatins are potent tubulin binding cytotoxic agents (colchicine domain MT inhibitors) of natural origin. They lead to antiangiogenesis and vascular disruption, resulting in tumor necrosis. Common structural features of combretastatins include a trimethoxylated ‘A’-ring, an often C3’ and C4’-substituted ‘B’-ring, and an ethene bridge in-between. Clinical trials with combretastatin A4-phosphate targeted, e.g., thyroid or ovarian carcinoma. Photoswitchable tubulin polymerization inhibitor combretastatin A4 (CA4) analogs (coined photostatins by the authors) were addressed by Thorn-Seshold and coworkers [101] in their well-documented study. As already for the parent drug, an activity difference was observed between the *E* and *Z* isomer [102]. It was logical to replace the central C=C bond with an N=N unit. Besides introducing the N=N unit, analogs with different substitution patterns were prepared (5 probes—e.g., **96**), as well as the azobenzene analogs of two nonspecific combretastatin prodrugs (e.g., **95**) and a hybrid probe with a biochemical targeting unit (**94**). Photoisomerization in PBS with UV/VIS monitoring showed a rapid and reversible process, without signs of degradation over hundreds of cycles (**93**: *E*→*Z* 388 nm, *Z*→*E* 508 nm, τ = 6 min—ranging between 0.8 and 120 min for other probes, depending on the substitution pattern). Illumination-dependent cytotoxicity was studied in cellular assays in MDA-MB-231 human breast cancer and HeLa cell lines, using frequently pulsed, short illuminations vs. dark conditions. The results showed a significant (~20–100-fold) increase in activity upon light exposure in both cell lines. Wavelength screening on HeLa cells (MTT assay) pointed toward the concentration of the isomer being the principal factor in the bioactivity (i.e., dose-response curves were in *Z* correlation with the relative *E*/*Z* ratios at the studied wavelengths). Further studies were conducted with **93** and its prodrug **95** in MDA-MB-231 cells, assaying cell membrane permeability (propidium iodide exclusion assay) and nuclear fragmentation (quantification of DNA content). A light-induced effect was observed in both cases, also corroborated by results gathered in Jurkat (T cell lymphoma) and HeLa cells (G2/M arrest: **93**-*Z*—500 nM, **93**-*E*—> 100 µM). Of note, using a rescue lighting regime (390 nm pulses alternating with a 515 nm pulse) decreasing the **93**-*Z* concentration effectively led to a reduced G2/M arrest (vs. normal cell cycle repartition under dark conditions). Examining PARP-cleavage (Western blot, HeLa cells), the PARP proteolytic signature typical of apoptosis was observed only after light-illumination. In an in vitro radioligand scintillation proximity assay, the competitive displacement of 3H-colchicine by **93** from its tubulin binding site was measured. For **93**-*Z* (390 nm), an EC_50_ = 30 µM was found, whereas the **93**-*E* showed no significant competitive binding to tubulin. In vitro tubulin polymerization inhibition was observed under 390 nm in a dose-dependent manner (EC_50_~5 µM vs. EC_50_(dark) >> 40 µM). Under 390 nm illumination regime, **93** led to dose-dependent microtubule depolymerization in cellulo vs. no effect under dark conditions (immunofluorescence imaging of endogenous tubulin). By imaging the end-binding protein EB3 (total number of EB3 comets, lifetime, speed, distance traveled), direct visualization of microtubule dynamics in live cells was undertaken while photoswitching **93** in situ (alternating 2 min phases of pulsed 405 nm and 514 nm light). Importantly, optical control over microtubule dynamics could be achieved with **93** also in vivo (mitotic progression of developing *C. elegans* embryo, ms pulses of 405 and/or 514 nm light) with single-cell precision. In mammalian tissue in vivo (cremaster muscle tissue of living mice), **93** illuminated at 390 nm had disruptive effects on the microtubule network that was not detected under dark conditions. Streu and coworkers used the same design for their [103] azo-combretastatin A4 (**93**) (i.e., central C=C to N=N) (Figure 17). Photoisomerization in CDCl_3_ (10 mM) with ^1^H NMR monitoring showed a maximum ~9/1 *Z*/*E* ratio under 10 min (400 nm LED flashlight). Photoisomerization at lower concentration was studied by UV/VIS monitoring (400 nm LED flashlight, 30 µM in DMSO, the maximum ratio reached in <10 s). The effect on tubulin polymerization in vitro was studied by turbidity assay. Under dark conditions (*E*), no activity was detected; however, the activity could be switched on upon light exposure (*Z*). Cytotoxicity of azo-combretastatin was studied in HeLa cells (MTT assay) under dark conditions (EC_50_ > 100 µM) and in the presence of light (10 s irradiation every 30 min with 400 nm LED flashlight; complete cell death at 0.5 µM), showing a more than 200-fold increase upon illumination. Parallelly, the light irradiation tolerance of the study system and CA4 was ascertained in control experiments. Azo-combretastatin (Azo-CA4—**93**) was also characterized by Hartman and coworkers [104]. Photoswitching was studied by UV/VIS, 380 nm irradiation (4.4 mW/cm^2^), providing the *Z* isomer in 30 s. The half-life was recorded in various solvents to rule out their effect, as longer periods than the previously described values were recorded, even under identical conditions (0.25% DMSO in H_2_O: 100 min, 0.25% DMSO in PBS: 88 min, 0.9% DMSO in PBS: 75 min, 10% ACN in PBS: 92 min, 20% ACN in PBS: 85 min). Alternating photoswitching and relaxation cycles could be operated over 24 h without significant degradation. In vitro inhibition of tubulin polymerization was studied in a fluorescent assay under dark and lit (pre-activated—380 nm, 4.4 mW/cm^2^, 1 min) conditions, showing a 2.8-fold difference between the isomers (**93**-*Z*: IC_50_ = 5.1 µM, CA4: IC_50_ = 1.9 µM). Cytotoxicity was assayed in HUVECs and MDA-MB-231 cell lines, using different illumination regimes. EC_50_ values ascertained a light activation and were in correlation with the pulse frequency, i.e., the concentration of the *Z* isomer (**93**-*Z*: EC_50_(MDA-MB-231) = 0.6 µM, EC_50_(HUVEC) = 0.4 µM; **93**-*E*: EC_50_(MDA-MB-231) = 21 µM, EC_50_(HUVEC) = 9.5 µM; CA4: EC_50_(MDA-MB-231) = 0.0021 µM, EC_50_(HUVEC) = 0.4 µM). As light irradiation did not restore the total activity of CA4, the stability of **93** toward GSH was studied. Both isomers degraded in the presence of GSH over the course of hours, leading to the formation of rearrangement and GSH-adduct products (LC-MS monitoring).

Brittain and coworkers set out to prepare a library of novel azo-combretastatins [105]. A total of 17 probes were prepared, with different (*O*-alkyl) substituents on the B-ring, applying previous SAR knowledge pointing toward alcohol or amine at the 3-positon being advantageous. Except for 4 *para*-phenol derivatives with extremely fast thermal relaxation, the other probes reached *E*→*Z* PSS within 3–7 min upon UV irradiation. Cell viability was screened on HeLa and H157 (lung cancer) cell lines (MTT assay) under dark and irradiated conditions (390–400 nm, 10 s pulse every 34 min). Of the 17 novel azo-combretastatins, 11 showed activity; however, significant light-dependence was observed for 3 probes with up to a 550-fold difference in activity. To obtain a better insight into the operation of **97**, tubulin binding was studied with ligand binding simulations; however, no significant difference was found compared to reference compound **93**. As another possible factor in bioactivity, aqueous solubility was studied [106]. Compound **97** is slightly less soluble than **93**, with a modest difference in *Z*/*E* solubility. Potential contribution to the effect of the longer *Z* half-life was also considered (^1^H NMR, water: **93**—46 h, **97**—138 h).

Thorn-Seshold and coworkers dedicated further studies to photoswitchable microtubule-targeting agents, with the aim of providing novel photoactivatable scaffolds (C=C bond based styrylbenzothiazoles—SBTs) to circumvent challenges with the broadly used azobenzenes (e.g., overlap of isomerization and assay imaging readout wavelengths, N=N reduction in cellular environments, suboptimal relaxation half-lives) [107]. Two novel CA4 analog probes (**98**, **99**) and their functional group permutation variants as negative controls were designed (coined SBTubs by the authors). The novel probes had no absorbance above 450 nm, showed convenient photoisomerization at 360 nm (PSS: ~85% *Z*), and suitable photoresistance under aerobic aqueous conditions. Thermal relaxation at 50–60 °C proceeded overnight (back photoisomerization unfeasible due to band overlap), therefore in a cellular context, *E*→*Z* switching could be envisaged. Of note, SBTubs photoswitching also operated with two-photon excitation (780 nm). Photo- and biochemical stability of the novel probes was ascertained by subjecting them to continuous high-power UV irradiation and GSH challenge assays, and preliminary metabolic assessment was performed (metabolism by liver microsomes, cytochrome inhibition, hERG binding). Light-dependent cellular antiproliferative effects were tested on HeLa cells (pulsed illuminations with 360 or 420 nm light, or dark conditions, 75 ms per 15 s, <1 mW/cm^2^; ~1–2 μM IC_50_ (~85% *Z*) vs. > > 20 μM IC_50_ (~100% *E*), no light-dependent activity for the negative controls). Mechanism of action was verified by inhibition of tubulin polymerization assay, target X-ray co-crystallization studies (*Z*-specific tubulin binding at the colchicine site), and imaging the MT network architecture in SBTub-treated cells (360 nm light). The latter showed similar dose dependency as the viability studies as well as a typical pattern of MT-inhibiting antimitotics (specificity corroborated with permutation variants and light-dependent G2/M phase cell cycle arrest for *Z*-SBTubs). Further conceptual studies were conducted with the better performing **99**. Notably, single isomerization in situ (18 s 360 nm low-power LED) resulted in a similar antiproliferative effect as that after repeated irradiations. In situ photocontrol (405 nm pulse) of MT dynamics could be operated orthogonally with GFP-EB3 (488 nm) or YFP-EB3 (514 nm) imaging. Temporal reversibility of the effect on MT dynamics was demonstrated by an EB3-GPF imaging assay, and its scope was extended to a further setup (A549 cell line).

To develop an alternative photoswitch unit, where *Z*- and *E*-bioactivities on the same target might straightforwardly be interchanged on demand by structural modifications depending on the planned application, Thorn-Seshold and coworkers studied hemithioindigo derivatives [108,109] in the context of microtubule-targeting antimitotic agents. Due to the breadth of available structural knowledge, the colchicinoid pharmacophore was selected as starting point. As between the pharmacophoric trimethoxyphenyl and methoxyphenyl rings, several types of linkers might be tolerated. The hemithioindigo unit was embedded into the bridge while keeping the colchicinoid substitution pattern to avoid loss of activity. Due to structural and synthetic considerations, four study structures were prepared (**100**–**103**), as well as three negative controls lacking specific on-target activity according to SAR considerations (having a modified substitution pattern) to rule out nonspecific effects in biological assays (novel probes coined “HOTubs” by the authors). Due to solubility reasons, photocharacterizations were conducted in a PBS/DMSO 25/75 mixture (450 nm irradiation giving the highest PSS content of the *E* isomer (65%), 530 nm irradiation the highest PSS content of the *Z* isomer (97%) –**103**, t_1/2_ = 30 min). The photoswitching was found to be repeatable (over 285 cycles) and reversible even in non-degassed solutions. Cellular antiproliferative activity was assayed on HeLa cells (MTT assay), applying a repeated illumination regime to keep a PSS equilibrium. Of the four novel derivatives, three showed feasible activity, and in line with the design reasonings, both dark-active and lit-active probes were obtained (lit-dark EC_50_ ratios ranging from 0.5 to 4.0). Importantly, the negative controls showed decreased and illumination-independent toxicity, suggesting cellular tolerability of the hemithioindigo structures. For gaining mechanistic insight into bioactivity, further studies were conducted. The effect on tubulin polymerization was assessed by immunofluorescence staining and confocal microscopy of the polymer microtubule network of the cells after extended treatment (24 h), confirming an illumination-dependent effect. Cell cycle repartition was assessed by flow cytometry, showing a G2/M cell cycle arrest typical of the pharmacological class in a dose- and illumination-dependent manner. In a follow-up study, further optimizations of hemithioindigo probes were addressed to improve the bioactivity while keeping the beneficial photochemical properties [110]. To this end, the substitution pattern was rationally designed, using a colchicine domain inhibitor indanocine scaffold as a starting point. A small set of novel probes with a *para*-methoxy group vs. the sulfur was prepared (coined HITubs by the authors) to allow subsequent SAR considerations. Compound **104** showed reversible photoswitching in polar aprotic solvents (*Z*→*E* 450 nm (~70% *E*), *E*→*Z* 530 nm (~97% *Z*)), a thermal half-life of ~40 s, and high fatigue resistance. Regardless of the pH, no photoswitching was detected in aqueous solution. Cellular studies were pursued, however, due to the expected biolocalization of the probes in cellular lipid environments. Cellular activity was assayed with resazurin antiproliferation assay on HeLa cells, the best performing probe showing an enhanced effect vs. previously disclosed HOTubs (**104**: EC_50_ dark (*Z*) = 0.11 µM, EC_50_ 450 nm (*E*) = 0.45 µM). To gain an insight into the mechanism, tubulin polymerization inhibition in a cell-free assay was addressed, giving an effect in the same range as that of colchicine for **104**. The effect on the cellular MT network was studied with immunofluorescence staining with confocal microscopy, showing a light-dependent action. Cell cycle repartition was studied by flow cytometry (propidium iodide incorporation), giving a further indication of tubulin binding.

In the realm of microtubule-stabilizing probes, with an aim to extend the potential biological applications (vs. microtubule destabilizers), Thorn-Seshold and coworkers developed photoswitchable PTX-based agents (coined “AzTax” by the authors) [111]. Based on structural and SAR considerations, the photoswitch unit (an azobenzene) was introduced at the 3’-amine so as to tune down the potency of PTX besides offering a substantial difference of activity between the isomers. Two structural factors were examined: the orientation of the azobenzene (*ortho*, *meta,* or *para* vs. the amide) affecting potency and substitution of the distal azobenzene aromatic ring affecting photochemistry. For the most potent series, further azobenzene substitution patterns were tested as well to check their effect on isomer potencies and solubility. Due to solubility reasons, photoswitching studies were conducted on diethanolamide models in aqueous media (PBS with <1% DMSO) (unsubstituted compounds: (PSS) 375 nm—80% *Z*, 410 nm—26% *Z*; τ~50 days; methoxylated compounds: (PSS) 375 nm—96% *Z*, 530 nm—11% *Z*; τ~24 h) with UV/VIS monitoring. Quantitative *E*→*Z* isomerization could be operated with overnight 60 °C heating of DMSO stock solutions. *Para*-amino compounds featured no observable photoswitching in aqueous conditions; however, in moderately polar environments (EtOAc), a reversible isomerization could be detected ((PSS) 410 nm—91% *Z*, 530 nm—21% *Z*; τ~11 min). Structure- and light-dependent cytotoxicity was assayed in HeLa cells (resazurin cell proliferation/viability assay) under dark or UV conditions. The best performing agent (**105**) was characterized with a 6-fold dark(all-*E*)/lit(mostly *Z*) EC_50_ ratio (EC_50_(dark) = 1.4 μM, EC_50_(360 nm) = 0.24 μM). Mechanism of action of **105** was further addressed in tubulin polymerization cell-free assays and studying the effect of the in situ irradiated probe on the cellular MT network (immunofluorescence imaging) and cell cycle repartition (flow cytometry). Spatiotemporal control over the activity with single-cell precision was demonstrated on HeLa cells expressing a fluorescent reporter (EB3-tdTomato) for polymerizing MTs (1 μM **105**, low-intensity 405 nm irradiation).

As a further antimitotic approach, targeting microtubule-associated motor proteins could be envisaged. In this respect, Tamaoki and coworkers designed a photoswitchable version for the selective centromere-associated protein E (CENP-E; kinesin-7) inhibitor GSK923295 (under clinical trials) disrupting the alignment of chromosomes during mitosis [112].

### 3.6. Photoswitchable F-Actin Modulating Agents

The migratory ability of cancer cells is critical for invading the adjacent tissues and the vasculature and for subsequent metastasis formation. In cell migration processes, cellular actin filaments and the actin cytoskeleton play a crucial role; therefore, the anticancer potential of cytoskeletal filament-directed agents is being studied (complementary to microtubule-based agents). Arndt and coworkers used a natural F-actin stabilizing product, jasplakinolide, as the starting point for their studies on the design of photoactivatable agents (coined optojasps by the authors) affecting the actine cytoskeleton [113]. Jasplakinolide was modified by an azobenzene photoswitch unit using an “azo-extension” approach. A set of eight different probes was designed, with varying amino acid side chain lengths and connection geometry of the photoswitch, besides a judicious selection of the azobenzene substitution pattern (targeting bidirectional photoswitching, appropriate solubility, and actin-selectivity). The novel probes showed efficient photoswitching (PSS: ~10:1 *Z*/*E*), appropriate thermal relaxation half-lives, with its range depending on the connection type (ACN, 25 °C: *ortho*: ~36 h, *para*: ~80 min; aq. buffer, 37 °C: *ortho*: ~200–250 min, *para*: ~18 min) and photostability (>12 h pulsed irradiations with 390 and 450 nm light). Light-dependent cellular cytotoxicity was assessed on HeLa cells. Optojasp-1 was found to be *Z*-active (EC_50_ (μM): *E* > 15, *Z* = 1.5) and was characterized in further studies. (Of note, *E*-active probes were identified as well among the novel optojasps). Regarding its effect on the cellular actin cytoskeleton (fluorescence microscopy), optojasp-1 led light-dependently (at 390 nm) to similar cellular phenotypes as that observed with known F-actin stabilizers, without affecting the MT network. With back-isomerization (475 nm irradiation) followed by dark incubation, the regular actin cytoskeleton could be phenotypically recovered. Target-selectivity (rate of F-actin polymerization) and lack of nonspecific interactions (due to solubility or aggregation issues) were ascertained. With optojasp-1, cell motility could be reduced in a light- and dose-dependent manner, and photocontrol over the completion of cell division could be achieved as well. The spatiotemporal control over cytoskeletal MRTF-A signaling was demonstrated in single-cell irradiation experiments.

### 3.7. Photoswitchable Metal Complexes

**Figure 18:**
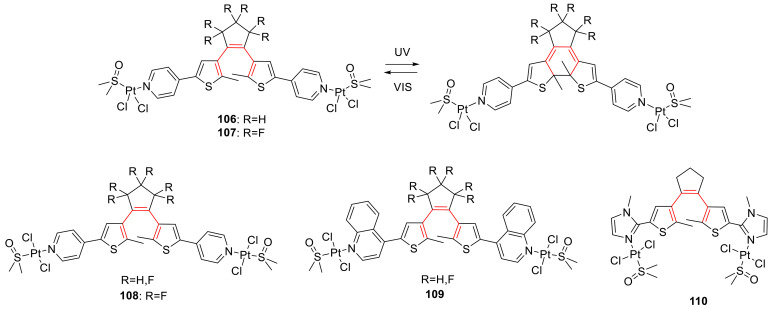
Photoswitchable metal complexes, the photoswitch unit indicated in red.

Gamez and coworkers designed photoswitchable platinum(II) complexes with a 1,2-dithienylethene unit in the ligand, with the aim of achieving distinct DNA-interacting and cytotoxic properties in the two isomeric forms (Figure 18) [114]. Two ligands were prepared with different substitution patterns on the central ring (H or F—**106**, **107**). Photoswitching of the novel ligands and the platinum complexes were studied by UV/VIS, indicating a reversible photocyclization. The closed forms were stable under dark conditions; however, they showed partial opening under daylight (24 h, in DCM solution, UV/VIS monitoring). DNA interactions were assessed by competitive binding studies (ct-DNA, ethidium bromide, fluorescence spectroscopy), and the DNA affinity of the complexes was quantified by the respective Stern–Volmer quenching constants. The closed forms showed an increased level of DNA interactions. The interaction with pBR322 DNA was studied by agarose gel electrophoresis, indicating significantly different behavior for the open and closed forms. Cell viability was studied on six cancer cell lines (A549 (lung adenocarcinoma), A375 (melanoma), DMS53 (small cell lung cancer, SCLC), GLC4 (SCLC), MCF7 (breast adenocarcinoma), PC3 (prostate adenocarcinoma)), first by a single-point assay with 50 µM complex. Complex **106** showed mostly >75% viability values both in the open and the closed form, with no significant differences between the isomers. For complex **107**, the open form had a modest effect (74–95%), whereas the closed form showed cytotoxicity on five of the studied cell lines (all except A549, 16–79%), together with a distinguishable activity of the isomeric forms (e.g., SCLC DMS53: 74% (open) vs. 29% (closed)). The single-point assay results were corroborated also by the determined IC_50_ values (48 h, **106**-open: IC_50_ = 77 µM, **106**-closed: IC_50_ = 76 µM, **107**-open: IC_50_ = 76 µM, **107**-closed: IC_50_ = 34 µM, *cisplatin*: IC_50_ = 8.53 µM). Exploiting the fluorescence of the complexes, cellular uptake was studied by confocal microscopy on four cell lines (affected—A375, DMS53, unaffected—PC3, A549). The results showed an efficient uptake for both complexes, however different staining in correlation with the cytotoxic properties.

In a follow-up study, Gamez and coworkers revisited the design of photoactivatable platinum(II) complexes using dithienylcyclopentene-based ligands, where light-induced changes occur in the coordinated ligand instead of the metal center itself [115]. Photoswitching of the ligands was characterized by UV/VIS and ^1^H NMR before pursuing platination. The interaction of the closed and open form of the novel complexes with pBR322 DNA was assayed by agarose gel electrophoresis, showing a structure- and concentration-dependent effect. Most of the closed isomers showed a DNA-unwinding ability, ascribed to insertion of the complexes and binding of the platinum atoms. In competitive binding studies (fluorescent intercalator—ethidium bromide—displacement assay), the closed forms had an increased quenching effect, therefore a better intercalating activity. Calculating the Stern–Volmer quenching constants, similar tendencies (open vs. closed forms) were observed for all the complexes studied. Cellular activity of the complexes was assessed on five cell lines: lung adenocarcinoma (A549), A375, MCF7, colorectal adenocarcinoma (SW620), and ovarian adenocarcinoma (SKOV3). Modest cytotoxic activities were detected in most cases (10 and 50 µM, above 75% survival), with no difference between the open and closed forms, except for the closed form of complex **108** and the open form of the photochemically inert (i.e., not cyclizable) complex **110** (**108**-open: IC_50_(A375) > 75 µM, IC_50_(SW620) > 75 µM; **108**-closed: IC_50_(A375) = 3.1 µM, IC_50_(SW620) = 2.4 µM; **110**: IC_50_(A375) = 3.4 µM, IC_50_(SW620) = 1.8 µM vs. *cisplatin*: IC_50_(A375) = 6.6 µM, IC_50_(SW620) = 13 µM). In the latter case, a different mode of action (vs. the other studied complexes) was assumed, which was confirmed by circular dichroism analysis of the interaction of the complexes with ct-DNA.

### 3.8. Photoswitchable Proteasome Inhibitors

**Figure 19:**
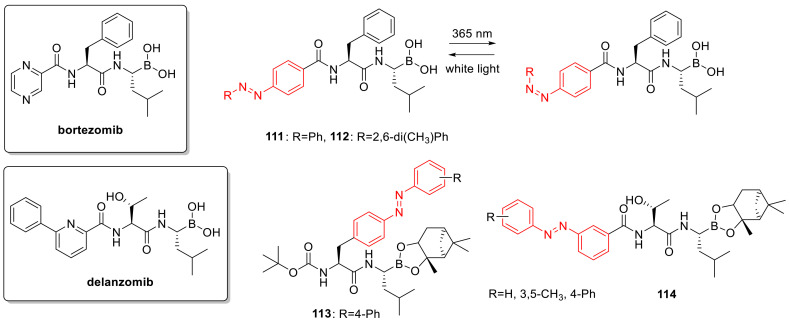
Photoswitchable proteasome inhibitors, the photoswitch unit indicated in red.

Proteasome inhibition via one/more active catalytic sites hampers the degradation of superfluous proteins (including abnormal and misfolded proteins) and could prevent the degradation of pro-apoptotic factors, on the other hand. The first in class *bortezomib* was approved in 2003 for multiple myeloma and mantle cell lymphoma. Its adverse effects include peripheral neuropathy and myelosuppression. Feringa and coworkers prepared photoswitchable proteasome inhibitors by adding an azobenzene unit to the peptidic part of the approved inhibitor *bortezomib*, based on structural and SAR information (Figure 19) [116]. The planar *E* form was expected to retain activity vs. the bent *Z* form. Photoisomerization and half-lives of the *Z* isomers were studied by UV and ^1^H NMR in DMSO-*d_6_*/D_2_O and H_2_O solutions (*Z*% upon irradiation: 72–90%, except **112**; half-life 37 °C (h): ~4–8 h). Reversible photoswitching (at least four cycles) was observed for all novel probes, also under reducing physiological conditions (**111** (20 µM), PBS (10 mM), pH 7.4, GSH (10 mM)). The biological activity of the inhibitors was studied in competition experiments on cell lysates before and after irradiation (365 nm). In accordance with the previous reasonings, activity was decreased upon irradiation (β1: 1.4–2.2-fold difference, β1i: 1.9–2.9-fold difference), with similar trends at β1 and β1i and a less pronounced difference on β5. Moreover, depending on the substitution pattern, differential activity and specificity for effect on active sites could be tuned. Cellular cytotoxicity assays were run on HeLa cells (MTT assay), showing between samples before and after irradiation (15 nm, 365 nm) minor differences in the IC_50_ values rather than in the curve shapes.

Blanco et al. also used the known agents *bortezomib* and *delanzomib* as starting points [117]. In one series, different azobenzenes were assessed at the site interacting with the P2 pocket of the target (*bortezomib* analogs **113**), whereas in a second series, different azobenzenes were introduced at the N-terminus (*delanzomib* analogs **114**). Photoisomerization and PSS composition were studied with ^1^H NMR in DMSO-*d_6_* solutions, showing significantly *Z*-enriched states upon irradiation (365 nm, 1 h) for most of the novel probes (*Z*%: 83–89 *bortezomib* analogs, 46–92 *delanzomib* analogs). In vitro activity was evaluated against β5 and β1 proteasome subunits in comparison with the parent drugs. Structural modifications were typically well tolerated; however, the highest difference observed upon irradiation was around 5-fold (decrease in activity). **113,** having the highest β5 activity besides a 5-fold difference between the isomeric states, was subjected to further cellular toxicity studies on HCT-116 colon colorectal and MDA-MB-468 breast carcinoma cell lines (7AAD assays). In cellular studies a reversal of activity was observed compared to the proteasome subunit assays (**113**-*E*: LD_50_(HCT-116) = 1.0 µM, **113**-*Z*: LD_50_(HCT-116) = 0.21 µM; **113**-*E*: LD_50_(MDA-MB-468) = 0.25 µM, **113**-*Z*: LD_50_(HCT-116) = 0.14 µM), **113**-*Z* showing modest cytotoxicity also on non-malignant MCF-10A cells, while the **113**-*E* tested inactive (till 5 µM).

### 3.9. Photoswitchable PROTACs

**Figure 20:**
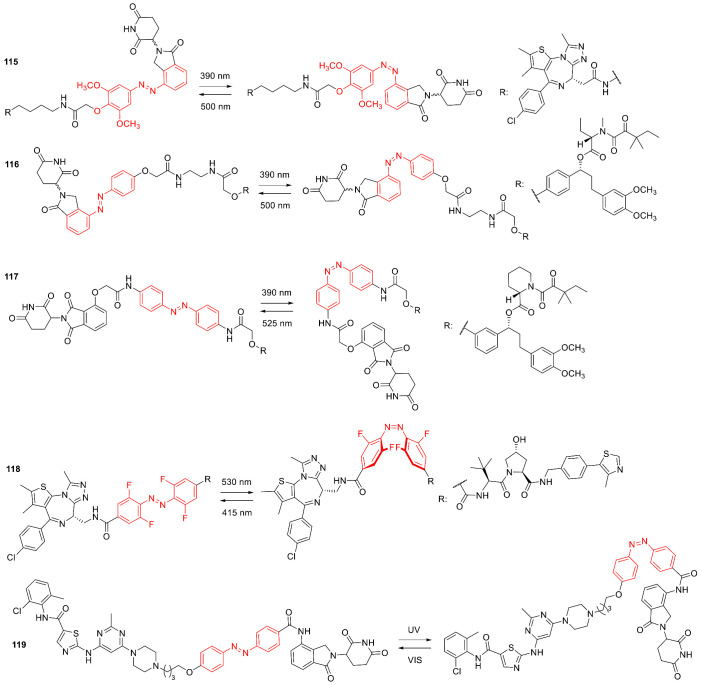
Photoswitchable PROTACs, with the photoswitch unit indicated in red.

The design of photoactivatable PROTACs was investigated by several groups. The construct of Reynders et al. consisted of a ligand for an E3 ubiquitin ligase (binding to the CRL4^CRBN^ complex), a photoswitch unit, and a ligand targeting BET family proteins (BRD2,3,4) or FKBP12. As CRBN ligands *pomalidomide* and *lenalidomide* were considered, whereas as photoswitchable linker either a conventional azobenzene or a diazocine (Figure 20) [118]. The first set of probes was prepared using a (+)-JQ1 ligand, which is an inhibitor of BRD2,3,4 and BRDT (13 novel probes, studying different substitution patterns and linker lengths). A second series targeting FKBP12 consisted of a CRBN-targeting glutarimide, an azobenzene photoswitch, a linker, and a synthetic ligand for FKBP12 (SLF) (6 novel probes studying different positions of the photoswitch). The lead compound of the first series (**115**) had >90% *Z* isomer in the PSS (390 nm), reverse isomerization at >450 nm providing >70% *E* form (thermal half-life (37 °C, DMSO): 8.8 h). The effect on cell viability was studied on RS4;11 lymphoblast cells under dark and light-irradiated conditions (100 ms 390 nm pulse every 10 s), confirming an increased cytotoxicity upon light irradiation (**115**: EC_50_ = 88 nM (390 nm), EC_50_ = 631 nM (dark)). Targeted protein (BRD2-4) degradation in RS4; 11 cells was studied by Western blot analysis under dark and light conditions with increasing concentrations of **115**, also confirmed in MB-MDA-231 and MB-MDA-468 cell lines. Light irradiation led to a decrease in BRD4 and BRD3 levels (100 nM–3 µM, higher concentrations: hook effect), not observed in the dark. A less pronounced effect was detected for BRD2. Further mechanistic studies confirmed the mode of operation of the novel probe (co-treatment with the CRL inhibitor MLN4924 fixing BRD2-4 levels upon irradiation, study following siRNA knockdown of CRBN, study following the methylation of glutarimide, effect on c-MYC levels, rescue experiment with thermal relaxation or optical inactivation, wavelength dependence of the effect). Time dependence of the BRD2-4 degradation and c-MYC down-regulation was assessed over 24 h. Following pulse irradiation also led to an increased rate of PARP-1 cleavage. Of the second series, **116** and **117** were selected for biological testing on RS4;11 cells. A light-dependent effect on FKBP12 degradation was observed, with a slower onset than for BRD2-4 (no protein between 6 and 12 h). The modular design approach of the studied probes could be a valuable starting point for the development of future photoactivatable PROTACs.

Carreira and coworkers used the BET-degrader ARV-771 (JQ-1 ligand for BRD4, VHL ligand for E3 ligase) as their starting point, designing a bistable, photoswitchable *ortho*-tetrafluoroazobenzene into the linker region (as a “pull-pull” diacid), the length of which is critical for activity (**118**) [119]. Based on structural considerations, the *Z* isomer was expected to exert protein degrader activity (i.e., have optimal distance between the warhead ligands). The photoisomerization in DMSO at 415 nm led to a PSS with 95% *E* isomer (Φ_ZE_(415 nm) = 0.65), the reverse process at 530 nm to a PSS with 68% *Z* isomer (Φ_EZ_(530 nm) = 0.28). No thermal isomerization of the *Z* isomer was observed over days in DMSO, ACN, or aq. buffer at 37 °C (i.e., the system is indeed bistable) and the probe was stable in the presence of glutathione (50 µM probe, 10 mM reduced glutathione, 3 days) and in cells as well (LC-MS analyses of cell lysates). Biological activity was tested on Ramos cells, with pre-irradiated (415 or 530 nm, 30 min) solutions of **118**. **118**-*E* led to BRD2 degradation in nanomolar concentration (6.5 h incubation), which was not detected for **118**-*Z* in the same range. Time-dependent degradation profiles (1–24 h) were recorded for the two isomers, showing an onset of effect for **118**-*E* at 3 h, reaching a maximum at 7 h and remaining constant in the following 17 h despite the single irradiation protocol. To ascertain the mechanism of action (proteasomal pathway), experiments with the NEDD8 inhibitor MLN-4924 were run. Interestingly, no significant degradation of BRD4 was observed, which points toward an altered selectivity profile vs. the parent ARV-771. To challenge the reversibility of the photoswitching, experiments with a two-step irradiation regime were run (single irradiation of 405 or 530 nm vs. two-step 405–530 or 530–405 nm irradiation), showing a reversal of activity after double irradiation. A similar reversal was obtained upon incubation of *E*-treated cells under 530 nm or *Z*-treated cells under 403 nm irradiation vs. dark conditions.

Jin et al. designed a photoactivatable PROTAC targeting BCR-ABL (implicated in chronic myelogenous leukemia) [120]. As E3 ligase ligand *lenalidomide* was used and the attachment point for the photoswitchable azobenzene unit was selected based on structural analysis (CRBN and *lenalidomide* complex X-ray structure). On the other side of the construct, the tyrosine kinase inhibitor *dasatinib* was conjugated. Upon assembly of the fully armed construct, different linker lengths were tested, compound **119** showing the best target protein degrading activity. Photoswitching was studied in DMSO solution (361 nm and white light, thermal half-life (25 °C, DMSO): 10.3 h), and stability against GSH reduction was ascertained as well. Cellular effects and selectivity were studied on BCR-ABL- (K562, cell proliferation assay—IC_50_ = 68 nm, cell viability assay—EC_50_ = 28 nm) vs. non-BCR-ABL-driven cell lines (A549, HCT116, MCF-7, HEK293T), showing a differential activity toward the BCR-ABL-driven cell line. The mechanism of action (CRBN-dependent ABL degradation) of compound **119** was examined in control experiments in the presence of *dasatinib*, an *N*-methylated version of compound **119**, *lenalidomide,* or MLN4924 (NEDD8-activating enzyme inhibitor). BCR-ABL degradation studies of the separate *E* and *Z* isomers confirmed a significant difference in activity, in line with previous structural analysis results (*E*: degradation of BCR-ABL and ABL at 100 nM, >90% BCR-ABL degradation after 32 h; *Z*: no BCR-ABL degradation till 500 nm, no reduction in BCR-ABL until 32 h), whereas ABL gene expression was not affected. The reversibility of action upon light irradiation following treatment with **119**-*E* was studied on K562 cells upon UVC irradiation regime vs. white light (VIS) conditions. BCR-ABL and ABL protein levels remained low in the VIS group vs. the UV-irradiated cells. On the other hand, **119**-*Z* treatment followed by VIS exposure also led to BCR-ABL degradation.

### 3.10. Photoswitchable Peptidomimetics

**Figure 21:**
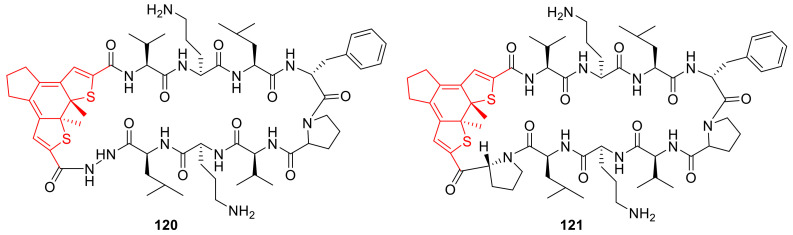
Photoswitchable gramicidin S analogs, the photoswitch unit indicated in red.

Synthetically modified peptides are opening novel avenues in chemical biology and medicinal chemistry and are a prime target for photopharmacology [121]. Albert et al. studied peptidomimetic photoswitchable histone methyltransferase inhibitors, targeting MLL1—an enzyme implicated in certain acute leukemias and solid tumors [122]. Komarov and coworkers prepared a photoswitchable derivative of the cyclic peptide gramicidin S (GS), using a diarylethene scaffold connected via a keto group (**120**, **121**) (Figure 21) [123]. In vitro cytotoxicity of the open and closed forms (**121**) was tested on HeLa, COLO-25 (colorectal cancer), and healthy MAEC (BALB/c mouse aortic endothelial) cell lines (MTT assay). The open form had IC_50_ values close to the parent GS (~6 µM) and a 5.5–8.0-fold higher cytotoxicity compared to the closed form. To move toward in vivo models, photoactivation in situ was planned, for which the irradiation conditions (wavelength, intensity) were determined in model tissues, trying to find an optimal compromise between photoisomerization efficiency, tissue penetration, and irradiation tolerability. As well, pharmacokinetic parameters were studied to ascertain stability (37 °C, human blood serum, 16 h) and reaching appropriate tumor tissue concentrations. For the latter, a mice model with Lewis lung carcinoma allograft was used. After intraperitoneal administration (5 mg/kg, single dose) of the closed form, blood and tumor tissues from sacrificed animals were studied by HPLC (different time points till 2 h). The highest blood concentration was reached in less than 15 min and maintained over 2 h, accompanied by an accumulation in the tumor tissue. Under dark conditions, over the 2 h period, less than 5–10% open form was detected in the blood samples. Upon VIS irradiation of the tumor areas (570 nm, 550 mW/cm^2^, 10–20 min), >75% close→open isomerization was observed, without significant change of the concentration of the open form in the blood (<12% conversion). In vivo antitumor effect was evaluated with the LLC model in C56B1/6 mice, with seven groups (8 animals/each) receiving daily i.p. injection of the vehicle (EtOH/saline 1/10), GS (1 mg/kg or 9.1 mg/kg) or the closed **121** (1.0 mg/kg or 9.1 mg/kg). **121**-treated groups were subjected to local VIS irradiation (100 mW/cm^2^, 15 min after injection) and compared with the unirradiated **121** treated control groups. Photo-treatment improved by 60% the survival compared to the control group, and post-mortem analysis revealed decreased and necrotic tumor tissues (~20 days).

Important in vivo information on the safety of photoswitchable anticancer peptides (**121** and its single-point mutants (LMB033, LMB037, LMB039, and LMB040) and their parent drug, gramicidin S) was obtained in a follow-up study by Komarov and coworkers [124]. The study peptides had a diarylethene photoswitch unit, therefore two thermally stable forms (open and closed, isomerization wavelengths: 325 and 653 nm). In previous studies, **121** had phototherapeutic index values (HC_50_ of the closed isomer (human erythrocytes) vs. IC_50_ of the open isomer) in the 8–11 range on different cancer cell lines, and the toxicity of its open form was similar to that of the parent drug, GS. To compare safety data, the LD_50_ values were determined in C57BL/6 for GS and both isomers of **121** as an indication of acute toxicity. The obtained results (LD_50_ (mg/kg)—**121**-open = 19, **121**-closed = 29, GS = 18) showed a decreased toxicity for the closed form upon IV administration (Cremophor EL/physiological saline 5/95 formulations); however, the difference was significantly lower than that observed in vitro (~1.5 vs. 20-fold). For a better understanding of the toxicity results, the pharmacokinetic parameters of the **121** isomers were measured in C57BL/6 mice. High plasma concentrations and slow clearance rates were observed, partly due to high plasma protein binding. Moreover, **121** had high metabolic stability under physiological conditions (blood plasma, microsomal). Both isomers had a significant inhibitory effect on CYP450 enzymes at 10 µM concentration. The distribution of the isomers following photoactivation was studied as a potential source of toxicity. The concentration of the isomers was measured in blood and tumor tissue samples prior to and after light irradiation (i.v.-administered **121**-closed mice with engrafted LLC), and a control experiment without irradiation was run. Following irradiation, the concentration of the open form increased in the tumor tissues but was not significant in the blood plasma. Of potential nonspecific interactions, hERG inhibition was assessed, showing an alarmingly high effect. To obtain peptides with more beneficial safety profiles, novel analogs were prepared and evaluated (LMB033, LMB037, LMB039, AND LMB040, structures not disclosed). High cytotoxicity was observed for the open forms on a series of human cancer cell lines and some interesting differences of activity (closed/open), particularly for LMB033 (17.5–60-fold). LMB033 and LMB040 had lower hemolytic activity as well, which translated to higher in vivo LD50 values in C57BL/6 mice (LD50 (mg/kg): 20–48). For LMB040, i.p. administration was less toxic than the i.v. route, which confirmed that the suboptimal pharmacokinetic parameters could be a reason for toxicity. As phototoxicity is a major concern for future therapeutic applications, LMB040 was tested in this sense on Balb/c mice. Following i.p. administration at high dose, the toxicity data of animals kept under normal daylight vs. in darkness was compared. The signs of toxicity observed underline the importance of tackling this issue in future development projects. Overall, despite an increased safety vs. the parent drug, in vitro assays were not sufficiently predictive for in vivo toxicity. On the other hand, efficient local activation with light could be achieved, and it did not lead to a rapid redistribution of the active isomer.

## 4. Challenges and Future Perspectives

Reversible or irreversible photoactivatable anticancer agents should comply with a plethora of photophysical, photochemical, pharmacokinetic, and pharmacodynamic criteria. For their successful development, contributions from distinct scientific fields are necessary, which might often be difficult with the present educational infrastructure, academic frameworks, and mindset. Many of the challenges that externally addressable drug delivery systems face are also relevant for the field of light-responsive drug molecules. Hoare and coworkers, in their study, identified tissue penetration, delivery to and retention at the active site, control over activation signal in vivo, materials and instrumentation complexity and translational models, and safety as the main issues to be addressed to facilitate translation toward therapeutic applications [125]. The wavelength required for the photoactivation and consequently the tissue penetration of the light trigger indeed is a bottleneck of future in vivo light-controlled approaches. In this respect, probes operating at higher wavelengths (red, infrared) or ideally in the biological window (λ = 650–1450 nm) [126] would be advantageous, and many efforts have been dedicated to developing such photoactivatable units of a specific design. In the field of azobenzene photoswitches, longer wavelength absorption generally leads also to faster thermal relaxation; moreover, the relative stabilities of the *Z* and *E* isomers could be altered as well. A typical approach for shifting the absorption toward higher wavelengths is to prepare push-pull systems with appropriately positioned electron-donating and electron-withdrawing groups (e.g., in certain azo dyes [127]). Not all structural modifications leading to higher wavelength absorption are feasible for photoswitches if they lead to a construct beyond the drug-like size or to a high number of rotatable bonds. In their 2015 account, Wooley and coworkers collected possible alternatives for the development of long-wavelength azobenzene photoswitches for in vivo applications [128]. For obtaining small-molecule azo-photoswitches with red-shifted absorption and slow (s/min) thermal relaxation in water, altering judiciously the substitution pattern might be a straightforward route. In this respect *ortho*-amino/fluoro [129] and tetra-*ortho*-methoxy/chloro/thioether substitutions were studied [130,131,132,133]. Moreover, azonium ions formed from variously methoxy-substituted derivatives showed interesting absorption properties as well [134]. Absorption could be shifted into the visible range with Lewis acid coordination of the azo group’s n-electrons (e.g., BF_2_) [135,136]. Diazocines (bridged azobenzenes) offer complete switching in both directions, a red-shifted absorbance, and a thermal relaxation rate in the minutes range [137,138]. However, due to the cyclic structure, the *Z* isomer is the more stable one, which is typically the less sought-for option. Hecht and coworkers reported a one-photon, strong donor-acceptor-based dihydropyrene photoswitch showing nearly quantitative (95%) isomerization at >800 nm [139]. In the field of photocages, examples of probes operating at long wavelengths, e.g., boron dipyrromethene (BODIPY) or heptamethine cyanine derivatives, could be cited [140,141,142,143,144].

Photoisomerization and photocleavage might profit from two-photon absorption that necessitates the use of femtosecond pulsed laser systems. This might be feasible by using specifically designed probes. Two-photon absorption (TPA) is a nonlinear optical process. The simultaneous absorption of two low-energy photons (occurring only at high light intensity) leads to photoreaction. Especially in the field of photocages, a wide variety of two-photon activatable probes are available, and work is continuously in progress for further derivatives with improved photophysical and photochemical profiles [145,146]. Dipolar TPA probes typically have electron-donating/acceptor groups attached to a central core by conjugated systems. Lengthening the conjugated system or altering the properties, positioning of substituents are general design principles, as well as preparing more complex constructs [147]. Next to modifications of the chromophore, specific formulations could also allow harnessing longer wavelength light, e.g., the use of upconverting nanoparticles or alternative sources of light, as the Cherenkov radiation (i.e., internal co-localization of the light source and the photoactive agent) [148].

Although a number of in vivo experiments on multicellular organisms are discussed in the above sections, typically, reports disclosing photoactivatable probes are based on in vitro assays (proof-of-concept studies). For future therapeutic applications, it would be highly needed to gain a better insight into how these systems could be operated under in vivo conditions. The most studied in vivo application of photoactivatable agents so far is vision restoration, a logical choice regarding the site of activation [149]. However, it demonstrates the applicability of the concept per se. For targeting tumors in deeper tissues, further obstacles still need to be overcome. Regarding in vivo photochemistry, Wooley and coworkers developed a fluorescence reporter to monitor in situ azobenzene photoswitching (in zebrafish, over 2 days) [150]. Feringa and coworkers classified potential therapeutic interventions from the point of view of how light-accessible the different organs are [151]. To reach deeper tissues, instrumentation developed for diagnostic purposes or PDT therapy could be exploited [152].

Besides releasing the active (form of the) drug, specifically designed photoactivatable systems could have further features. Notably, as also illustrated by some examples in the previous sections, photoresponsive probes could also offer in situ monitoring of the drug release (i.e., rational dosimetry). Typically for photocages, release or formation of a fluorescent species is a general approach for real-time monitoring of the photorelease. Particularly when moving toward in vivo systems, getting quantitative information on free drug concentration has tremendous practical importance. Particularly in the realm of nanodevices, light-triggered action could be complemented with imaging modalities (i.e., theranostic construct designs) [153,154,155].

On-target activity requirements for photocages and photoswitches differ substantially. Photocages rely absolutely on the activity of the released compound, i.e., native ligand, which is usually a clinically used drug. Photocaging approach, however, also offers a possibility to use more potent (toxic) compounds, which cannot be used directly due to side effects or unsuitable physicochemical and pharmacokinetic properties. Additionally, the introduction of PPGs enables tuning drug-likeness of the prodrug. As PPGs must fulfill many requirements, there are not available PPGs with robust properties, which would allow for tuning fine properties (solubility, permeability, etc.) of photocaged compounds. This is a substantially unexplored area, which might offer significant improvements in the coming years. The on-target activity of photoswitches is a considerably more demanding task. Usually, photoswitchable moieties are relatively big and might substantially impair intrinsic binding. Nevertheless, the herein presented structures demonstrate that it is a feasible task. At this point, photoswitchable PROTACs are specific and successful examples offering a general approach to locating photoswitchable moieties as linker units or locating photoswitchable moieties on E3 ligase recruiting ligand and not interfering with the protein recruiting part.

## 5. Conclusions

Photocaging and photoswitching are known approaches as alternatives in the discovery of novel anticancer compounds. As the discovery of anticancer compounds (or novel APIs themselves) is a highly demanding task, adding another level of complexity raises several further concerns, such as regulatory issues, different pharmacokinetic properties of the isomers, the dependence of the photochemical properties on the environment to name just a few. Therefore, it makes it fully understandable that many reports at present are coming from academic groups describing the idea and very basic implementation. Currently, we are in the phase where the concept is being developed on the level of compounds and is focused on optimizing compound properties, while biological activity is mainly validated with cancer cell line assays. Rapid development and constantly improved compounds are leading researchers into the stage that compounds will be evaluated in animal models, and proposed improvements in anticancer therapy will be thus validated. There are still many potentials for novel concepts to be explored, and photocaging and photoswitching are undoubtedly some of the most exciting areas of anticancer research.
